# Different nitrogen sources speed recovery from corallivory and uniquely alter the microbiome of a reef-building coral

**DOI:** 10.7717/peerj.8056

**Published:** 2019-11-15

**Authors:** Mallory M. Rice, Rebecca L. Maher, Rebecca Vega Thurber, Deron E. Burkepile

**Affiliations:** 1 Department of Ecology, Evolution, and Marine Biology, University of California, Santa Barbara, Santa Barbara, CA, USA; 2 Department of Microbiology, Oregon State University, Corvallis, OR, USA; 3 Marine Science Institute, University of California, Santa Barbara, Santa Barbara, CA, USA

**Keywords:** Corallivory, Seawater warming, Nitrate, Ammonium, Coral microbiome, Nutrient loading

## Abstract

Corals are in decline worldwide due to local anthropogenic stressors, such as nutrient loading, and global stressors, such as ocean warming. Anthropogenic nutrient loading, which is often rich in nitrate, inhibits coral growth and worsens corals’ response to warming while natural sources of nitrogen, such as ammonium from fish excretion, promotes coral growth. Although the effects of nutrient loading and ocean warming have been well-studied, it remains unclear how these factors may interact with biotic processes, such as corallivory, to alter coral health and the coral microbiome. This study examined how nitrate vs. ammonium enrichment altered the effects of increased seawater temperature and simulated parrotfish corallivory on the health of *Pocillopora meandrina* and its microbial community. We tested the effects of nitrogen source on the response to corallivory under contrasting temperatures (control: 26 °C, warming: 29 °C) in a factorial mesocosm experiment in Moorea, French Polynesia. Corals were able to maintain growth rates despite simultaneous stressors. Seawater warming suppressed wound healing rates by nearly 66%. However, both ammonium and nitrate enrichment counteracted the effect of higher temperatures on would healing rates. Elevated seawater temperature and ammonium enrichment independently increased Symbiodiniaceae densities relative to controls, yet there was no effect of nitrate enrichment on algal symbiont densities. Microbiome variability increased with the addition of nitrate or ammonium. Moreover, microbial indicator analysis showed that Desulfovibrionaceae Operational taxonomic units (OTUs) are indicators of exclusively temperature stress while Rhodobacteraceae and Saprospiraceae OTUs were indicators of high temperature, wounding, and nitrogen enrichment. Overall, our results suggest that nitrogen source may not alter the response of the coral host to simultaneous stressors, but that the associated microbial community may be distinct depending on the source of enrichment.

## Introduction

Corals typically thrive in ecosystems with oligotrophic waters due to tight nutrient cycling between corals and algal symbionts (Muscatine & Porter, 1977). However, anthropogenic nutrient pollution has increased nutrient availability on many reefs worldwide, especially with respect to nitrogen (N) availability (D’Angelo & Wiedenmann, 2014; Fabricius, 2011). Anthropogenic-derived sources of N, often in the form of nitrate, have deleterious consequences on coral growth and physiology (D’Angelo & Wiedenmann, 2014; Shantz & Burkepile, 2014) and can make corals more susceptible to seawater warming (Burkepile et al., 2019; Fabricius et al., 2013; Vega Thurber et al., 2014). These patterns are concerning given that seawater warming events have been increasing in frequency and intensity due to global climate change (Hughes et al., 2018).

Alternatively, natural sources of N (e.g., ammonium from fish excretion) can benefit corals by increasing coral growth and calcification (Holbrook et al., 2008; Meyer & Schultz, 1985; Meyer, Schultz & Helfman, 1984; Shantz & Burkepile, 2014). In fact, corals under ammonium enrichment receive more translocated carbon from Symbiodiniaceae (formerly *Symbiodinium*; LaJeunesse et al., 2018) than corals enriched with nitrate (Ezzat et al., 2015). Ammonium can even alleviate the negative impacts of seawater warming by maintaining the response mechanisms of coral immunity at the molecular level (Zhou et al., 2017). Indeed, anthropogenic- vs. naturally occurring N seem to have contrasting impacts on coral physiology and susceptibility to seawater warming (Burkepile et al., 2019). Yet these effects on physiology can vary with N concentration (Ferrier-Pages et al., 2000; Marubini & Davies, 1996; Marubini & Thake, 1999), which likely influences how corals respond to other stressors (Fabricius et al., 2013).

Coral predation (i.e., corallivory) is a common biotic process on reefs with many corallivores removing coral mucus, tissue, and skeletal structure. Scraping and excavating corallivory by parrotfishes and pufferfishes removes coral tissue and varying degrees of skeletal structure, which can substantially reduce coral growth rates (Cole, Pratchett & Jones, 2008; Rice, Ezzat & Burkepile, 2019; Rotjan & Lewis, 2008). This impact can exacerbate corals’ response to concurrent stressors and even prevent recovery from anthropogenic perturbations (Rice, Ezzat & Burkepile, 2019). For instance, parrotfish corallivory inhibited the recovery of *Orbicella* spp. colonies after a bleaching event (Rotjan et al., 2006). Nutrient loading can also interact with fish corallivory to drive changes in coral mortality. In the Florida Keys, parrotfish corallivory increased *Porites* mortality by 62% when corals were simultaneously exposed to anthropogenic nutrient sources (Zaneveld et al., 2016). These patterns may be driven by increases in opportunistic bacteria and wounding driving changes in the coral microbiome directly (Zaneveld et al., 2016) or resulting from compromised host immunity and thus inability of the coral to regulate its microbiome (Zaneveld, McMinds & Vega Thurber, 2017).

The coral microbiome is dominated by bacteria, protozoans, and archaea that perform a multitude of functions from nutrient cycling to protecting the host against opportunistic bacteria (Bang et al., 2018; Bourne, Morrow & Webster, 2016; Ritchie, 2006; Rohwer et al., 2002). Some commensal microbes produce antibacterial compounds to prevent opportunistic bacteria from colonizing the host (Bourne, Morrow & Webster, 2016; Ritchie, 2006). When the microbial community is disturbed, populations of opportunistic bacteria can become established, which can compromise the holobiont immunity and lead to coral mortality (Glasl, Herndl & Frade, 2016). This mechanism has been proposed for *Porites* corals under simultaneous thermal stress and nutrient enrichment, and even parrotfish corallivory (Zaneveld et al., 2016). Yet, commensal microbes can help corals resist and recover from abiotic stress and are critical for enabling their host to cope with challenging environmental conditions (Bang et al., 2018; Bourne, Morrow & Webster, 2016). In a previous analysis of the coral microbiomes included in this study, we demonstrated that stressors primarily act additively or antagonistically, not synergistically, to alter microbial community composition with high temperature and simulated corallivory wounding independently causing the strongest responses (Maher et al., 2019). Additionally, we showed that changes in community structure with stress are driven by increases in opportunistic taxa, rather than the depletion of symbionts. When considering holobiont health, it is vital to understand how the interactions between corals and their microbial counterparts respond to anthropogenic forcing (Rädecker et al., 2015; for review see McDevitt-Irwin et al., 2017).

It remains unclear how N source (nitrate vs. ammonium) may alter corals’ response to elevated temperatures and corallivory. To that end, our study seeks to evaluate how different N sources (nitrate vs. ammonium) may mediate changes in: (1) coral growth rates, (2) wound healing rates, (3) Symbiodiniaceae densities, and (4) bacterial community dynamics in response to concurrent seawater warming and corallivory. We hypothesized that nitrogen source would differentially mediate the effects of seawater warming and simulated corallivory. Further, we predicted that varying nitrogen source would produce distinct microbial communities with indicator taxa that suggest potential functional responses to multiple stressor regimes.

## Materials and Methods

### Study species

We used a full factorial mesocosm experiment to test how nitrogen source (nitrate vs. ammonium) may alter a coral’s response to seawater warming and corallivory. The experiment was conducted at the Richard B. Gump South Pacific Research Station in Moorea, French Polynesia (17°29′26.04″S, 149°49′35.10″W). Research was completed under permits issued by the French Polynesian Government and the Haut-commissariat de la République en Polynésie Francaise (Protocole d’Accueil 2005–2018). *Pocillopora meandrina* was chosen as the study species because it is one of the most abundant corals on the fore reef in Moorea (Edmunds, 2018) and is heavily preyed on by parrotfishes in the Pacific (Cole, Pratchett & Jones, 2008). We distinguished this taxon according to its distinct morphology, although we acknowledge that definitive taxonomy of *Pocillopora* spp. is challenging in this region (Edmunds et al., 2016).

### Experimental design

In September of 2016, the experiment was conducted in twelve independent 150 L flow-through, temperature controlled mesocosms on a 12:12 light:dark cycle (Aqua Illumination Hydra 52 LEDS) at ~700 mmol m^2^ s^−1^. Seawater was pumped from Cook’s Bay and filtered with a 20 μm sediment filter before entering the mesocosms. A total of 10 healthy *P. meandrina* colonies with no observed corallivory were collected at three to four m depth on the north shore fore reef and transported in seawater by boat to the Gump Research Station. A total of 12 nubbins (2.7 ± 0.05 cm height) were fragmented from each colony and epoxied (using Z-spar A-788) onto one cm^2^ plastic mesh. Nubbins were allowed to recover for ~24 h in the mesocosms at ambient temperatures (26 ± 1 °C).

After the acclimation period, half of the coral nubbins were mechanically injured on the branch tip using eight mm snub nose pliers to mimic parrotfish bites. The pliers were sterilized with ethanol and heat after each nubbin to prevent the transfer of microbes across replicates. The injuries were 45.2 ± 1.5 mm^2^ and ~2 mm deep, resulting in a single wound that removed the tissue layer and some skeletal structure. The injuries resembled a wound similar to a scraping parrotfish bite. Coral nubbins were randomly assigned to the following treatment tanks (*n* = 2 tanks per treatment combination): (1) 26 °C, (2) 29 °C, (3) ammonium and 26 °C, (4) nitrate and 26 °C, (5) ammonium and 29 °C, and (6) nitrate and 29 °C. Five intact and five wounded coral nubbins were in each tank (*n* = 10 nubbins per treatment; Fig. S1), and there were no differences in the initial weight (29.1 ± 0.7 g) of the coral nubbins across treatments (Kruskal–Wallis; χ^2^ = 8.2, *P* = 0.7).

After ~24 h of acclimation, half of the mesocosms were gradually raised from 26 to 29 °C, over a 24-h period (~1 °C change per 8 h), to reach temperatures observed during summer seawater warming in Moorea (Pratchett et al., 2013). To establish the pulse nitrogen treatments, the mesocosms assigned to nitrogen treatments were enriched every ~12 h to 4 µM NO_−_^3^ or 4 µM NH_+_^4^ from stock solutions of KNO_3_ and NH_4_Cl, respectively. Background nutrient concentrations in the seawater system during this time period were 0.34 µM NO_−_^3^, 0.21 µM NH_+_^4^, and 0.15 µM soluble reactive phosphorus. The flow to tanks was ceased for 1 h during the enrichment. Nubbins were haphazardly moved within the tank every 2 days to avoid position effects. The experiment was maintained for 21 days.

### Coral growth rates, Symbiodiniaceae densities, and wound healing rates

At the beginning and end of the experiment, coral nubbins were buoyant weighed to determine changes in mass for growth rates (Davies, 1989; Jokiel, Maragos & Frankzisket, 1978). For measurements of wound healing rate, initial and final photos were taken using an Olympus TG-4 camera and ruler and processed in ImageJ for scar area. A wound was considered healed if there were visible polyps in the wound area. After the experiment, coral nubbins were frozen at −40 °C for microbiome analysis. From each of the 12 treatments, six nubbins were randomly selected for microbial analysis while controlling for parent colony and tank effects. The tip of each nubbin was clipped off using sterilized bone cutters, and frozen at −80 °C until DNA extractions. The remaining coral tissue was removed using 0.7 µm filtered seawater (FSW) and an air brush and collected into Falcon tubes. The tubes were centrifuged at 3,000 rpm for 10 min. The supernatant was removed and the Symbiodiniaceae pellet was resuspended with 10 mL of 0.7 µm FSW. Symbiodiniaceae densities were quantified using compound microscopy and a hemocytometer (*n* = 4 counts per replicate). The coral skeletons were dried at 60 °C for 7 days, allowed to cool to room temperature, and wax-dipped at 60 °C to determine surface area by regressing the difference in weight between single and double wax dippings against the surface area of known objects (Stimson & Kinzie, 1991). Growth rates and Symbiodiniaceae densities were normalized by the surface area of each nubbin.

### 16S library preparation, sequencing, and initial data processing

DNA was extracted from 72 samples (*n* = 6 per treatment) representing a subset of the experiment using the MoBio Powersoil^®^ DNA Isolation Kit. Amplicon libraries were prepared for the V4 region of the 16S rRNA gene using the primer pair 515F (5′-GTG CCA GCM GCC GCG GTA A-3′) and 806Rb (5′-GGA CTA CHV GGG TWT CTA AT-3′) that targets bacterial and archaeal communities (Apprill et al., 2015; Parada, Needham & Fuhrman, 2016). Amplicons were barcoded with Schloss-indexed barcoding primers with Nextera adapters, pooled in equal volumes for sequencing (Kozich et al., 2013) and purified with AMPure XP beads. Paired-end sequencing was performed on the Illumina MiSeq platform, 2 × 300 bp end version 3 chemistry according to the manufacturer’s specifications at the Oregon State University’s Center for Genome Research and Biocomputing Core Laboratories.

QIIME (v1.9) (Caporaso et al., 2010b) was used to process all 16S sequence libraries. Demultiplexed raw reads were trimmed and pair-end sequences merged. Chimeric sequences and sequences with a total expected error of >1 for all bases were discarded. 97%-similarity operational taxonomic units (OTUs) were picked using USEARCH 6.1 (Edgar, 2010), QIIME’s subsampled open-reference OTU-picking protocol (Rideout et al., 2014), and the 97% GreenGenes 13_8 reference database (McDonald et al., 2012) to create a starting OTU table. Taxonomy was assigned using UCLUST, and reads were aligned against the GreenGenes database using PyNAST (Caporaso et al., 2010a). The aligned reads were then used to reconstruct a phylogenetic tree using FastTreeMP (Price, Dehal & Arkin, 2010).

Operational taxonomic units were removed if their representative sequences failed to align with PyNAST to the GreenGenes database or if they were annotated as mitochondria or chloroplasts. After this step, the OTU table had 3,383 unique OTUs, and the number of reads per sample ranged from 1 to 87,262 with a median of 9,742 per sample. OTUs with less than 100 reads across the table were removed resulting in a total of 430 unique OTUs. We did not find that any low count OTUs were associated with one particular sample. After these quality control steps, ten samples were found to contain fewer than 1,000 reads and were thus removed from the dataset (Table S1).

In *R* (v3.4.0) the package *phyloseq* (v1.20.1) (McMurdie & Holmes, 2013) was used to rarefy the resulting table to exactly 1,070 sequences per sample, and to calculate from this rarefied table beta diversity metrics including Bray Curtis, Binary Jaccard, Weighted Unifrac, and Unweighted UniFrac dissimilarities. For beta diversity metrics, the OTU table was first log-transformed in *phyloseq*. Inclusion of all four distance measures allows for a robust analysis of community dynamics including ecological and phylogenetic changes in bacterial abundance (Bray Curtis and Weighted Unifrac) and in the presence or absence of certain bacterial species (Binary Jaccard and Unweighted Unifrac). Also from this rarefied table, alpha diversity metrics including Faith’s phylogenetic diversity (Faith, 1992), Chao1 statistic (Chao & Chiu, 2016), and Simpson’s diversity index (Heip, Herman & Soetaert, 1998) were calculated in *phyloseq*.

### Growth rates, wound healing, and Symbiodiniaceae densities data analysis

All data analysis was conducted in R (v3.4.3) (R Development Core Team, 2017) and all figures were produced using *ggplot2* (Wickham & Wickham, 2009). Treatment effects on coral growth and Symbiodiniaceae densities were assessed with linear mixed-effects models (LMMs) with the *lmer* function in R (Bates et al., 2015) with temperature, nitrogen, wounding, and the interactions as fixed effects and tank and parent colony as random effects. Wound healing rates were analyzed similarly but with temperature, nitrogen, and their interaction as fixed effects and tank and colony as random effects. Random effects were dropped if not significant in the model according to Chi-squared tests, resulting in final LMMs that have the most parsimonious random effects structure (Zuur et al., 2009). For all models, only colony was a significant random effect, thus tank was excluded from all final models. Model residuals were visually assessed for Gaussian distribution and homoscedasticity. The significance of fixed effects was determined using the *anova* function from the *lmerTest* package with Kenward-Roger correction for degrees of freedom (Zuur et al., 2009). Multiple comparisons were done with least-squares means using the *lsmeans* function (Lenth, 2016). An outlier in growth rate (6.93 mg cm^−2^ day^−1^) was removed from the analysis because it was >1.5 larger than the interquartile range of the data. The removal of the outlier did not change the results or interpretation.

### Microbial community data analysis

We previously evaluated how these individual and multiple stresses affected bacterial community taxonomic composition, evenness, and diversity (for details see Maher et al., 2019). In this study, however, microbial analyses were focused to investigate potential microbiome-dependent mechanisms underlying significant changes in host responses to the treatments and the bacterial community response to differences in nitrogen regimes, two aspects that were not explored in the previous study. Microbial community alpha and beta diversity were evaluated for associations with host responses (i.e., growth rates, wound healing rates, Symbiodiniaceae densities). First, alpha diversity metrics were regressed against host responses using LMMs with host response as the fixed effect and tank and parent colony as random effects. Next, associations between microbial community beta diversity and host responses were assessed with a PERMANOVA using the *adonis* function in the package *vegan* (v2.4.3) (Oksanen et al., 2007) for each of the four beta diversity metrics. Associations between microbial community beta diversity of wounded corals and treatment effects of temperature, nitrogen, and the interaction were then assessed with *adonis*. Homogeneity of group dispersions for wounded corals was independently assessed for temperature, nitrogen source, colony, and tank with PERMDISP using the *betadisper* function in the package *vegan* (Oksanen et al., 2007). Significant results were ordinated and visualized using NMDS in *phyloseq*. The core microbiome was evaluated from a relative abundance, unrarefied table with the package *microbiome* (v1.5.31) and defined as those taxa present in ≥50% of samples (Lahti et al., 2017).

Taxa indicative of any treatment combination (e.g., ammonium enriched and wounded under ambient temperature) were investigated using indicator species analysis (De Caceres & Legendre, 2009; De Caceres, Legendre & Moretti, 2010). Indicator species analysis involves calculating an indicator value between a species and each group that reflects both the exclusivity, occurring only in a single treatment group, and fidelity, occurring in all samples of a treatment group. The rarefied OTU table was used in the function *multipatt* from the package *indicspecies* (v1.7.6) (De Caceres & Legendre, 2009; De Caceres, Legendre & Moretti, 2010; Dufrêne & Legendre, 1997). The *multipatt* function identifies species that are associated with a particular treatment group by calculating an Indicator Value index with a correction for unequal group sizes using the function *IndVal.g*.

## Results

### Growth rates, wound healing rates, and Symbiodiniaceae densities

**Figure 1:**
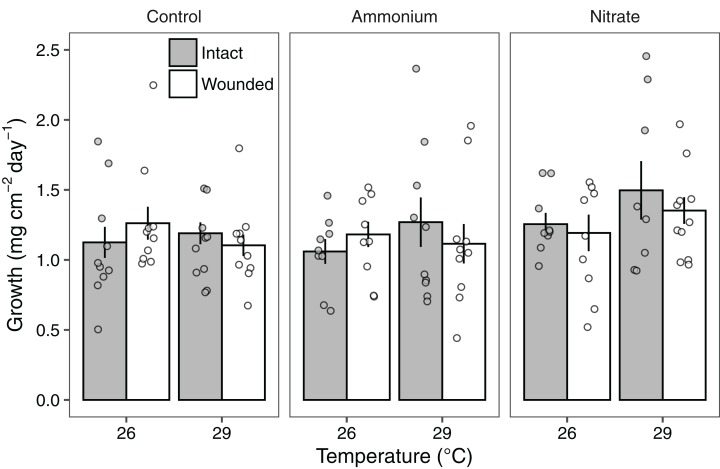
The growth rates of *Pocillopora meandrina* corals across treatments. Growth rates (mg cm^−2^ day^−1^; mean ± SE) of intact (gray bars) and wounded (white bars) *Pocillopora meandrina* nubbins under temperature (26 °C, 29 °C) and nutrient (control, ammonium, nitrate) treatments. The points show the distribution of the data.

**Figure 2:**
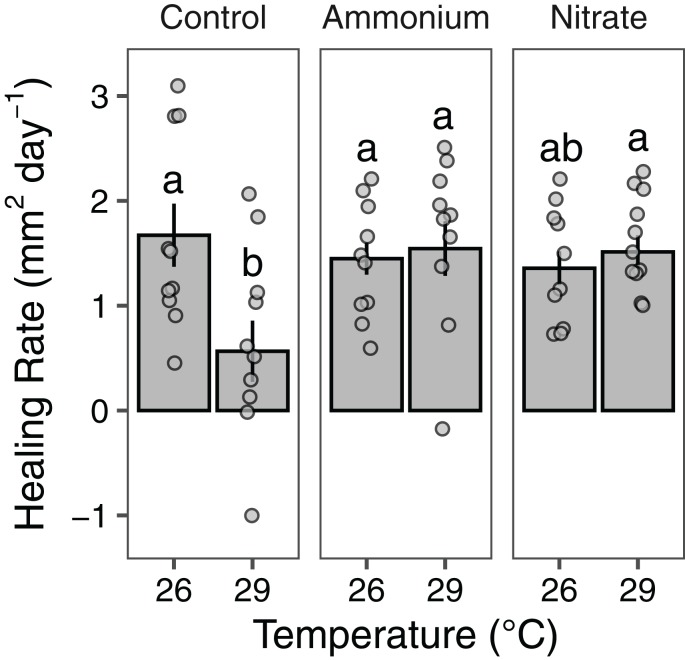
Wound healing rates of *Pocillopora meandrina*. The wound healing rates (mm^2^ day^−1^; mean ± SE) of wounded *Pocillopora meandrina* nubbins under different temperature and nutrient treatments. The points show the distribution of the data. Letters represent significant difference among treatments.

Growth rates of individual *P. meandrina* nubbins ranged from 0.49 to 2.38 mg cm^−2^ day^−1^. We did not observe main effects or interactions among seawater warming, nitrogen source, or simulated wounding on *P. meandrina* growth rates (Fig. 1; Table S2). For wound healing, there were no main effects of temperature (LMM; *F* = 3.57, *P* = 0.065) or nitrogen enrichment (LMM; *F* = 2.09, *P* = 0.14), yet there was a significant interaction between temperature and nitrogen enrichment (LMM; *F* = 6.51, *P* < 0.01; Fig. 2; Table 1). Pairwise comparisons revealed that healing rates were reduced ~66% at 29 °C compared to 26 °C under ambient nutrient conditions (*P* < 0.01; Table S3). At 26 °C, coral nubbins exposed to ammonium enrichment had faster wound healing rates than controls at 29 °C (*P* < 0.05; Table S3), but coral nubbins exposed to nitrate at 26 °C did not (*P* = 0.063; Table S3). For corals at 29 °C, nitrogen enrichment removed the negative effect of seawater warming on healing rate regardless of nitrogen source. When comparing the wound healing rates at 29 °C, we found that both ammonium and nitrogen enrichment increased healing rates by ~63% compared to ambient conditions (*P* < 0.05; Table S3). However, there were no differences in the wound healing rates for *P. meandrina* nubbins enriched with either ammonium or nitrate across temperature treatments (*P* > 0.9 for all comparisons; Table S3).

**Table 1:** Linear mixed-effects model results for healing rate (mm^2^ day^−1^) of wounded corals with Kenward-Roger approximation for degrees of freedom.

Fixed effects	*df*	*F*	*P*
Temperature	1	3.566	0.0653
Nutrient	2	2.091	0.135
Temperature × Nutrient	2	6.505	**<0.01**

**Note:**

*P*-values defined as significant at a threshold of 0.05 are highlighted in bold.

**Figure 3:**
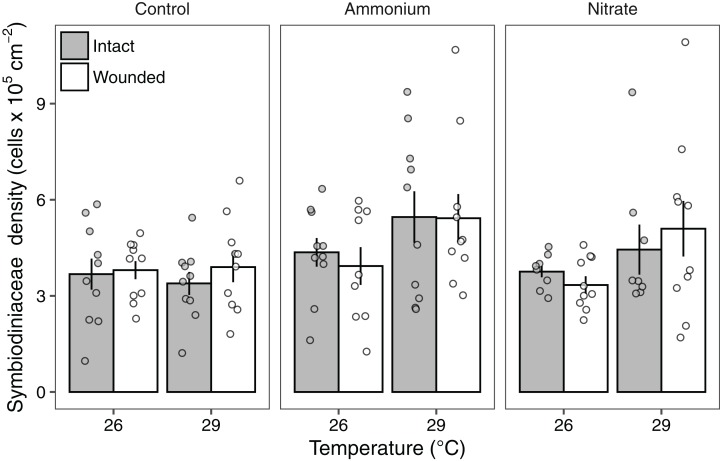
Symbiodiniaceae densities of *Pocillopora meandrina* corals across treatments. Symbiodiniaceae densities (10^5^ cells cm^−2^; mean ± SE) of intact (gray bars) and wounded (white bars) *Pocillopora meandrina* nubbins under temperature (26, 29 °C) and nutrient (control, ammonium, nitrate) treatments. The points show the distribution of the data.

Symbiodiniaceae densities ranged from 1.03 to 10.7 × 10^5^ cells cm^−2^. Seawater warming increased Symbiodiniaceae density (LMM; *F* = 7.91, *P* < 0.01; Fig. 3; Table 2). There was also a significant effect of nitrogen source (LMM; *F* = 4.55, *P* < 0.05; Fig. 3; Table 2) with ammonium enrichment increasing Symbiodiniaceae densities by 30% relative to control conditions (*P* < 0.01; Table S4). We did not observe differences in Symbiodiniaceae densities for corals enriched with nitrate relative to controls (*P* = 0.51; Table S4) or nitrate relative to ammonium enrichment (*P* = 0.18; Table S4). Further, there was no interaction between seawater warming and nitrogen source on Symbiodiniaceae densities (LMM; *F* = 2.52, *P* = 0.086; Fig. 3; Table 2). Simulated corallivory had no effects on Symbiodiniaceae densities and there also was no three-way interaction between temperature, nitrogen, and wounded treatments on Symbiodiniaceae densities (Fig. 3; Table 2).

**Table 2:** Linear mixed-effects model results for Symbiodiniaceae densities (10^5^ cells cm^−2^) with Kenward-Roger approximation for degrees of freedom.

Fixed effects	*df*	*F*	*P*
Temperature	1	7.909	**<0.01**
Nutrient	2	4.545	**<0.05**
Wounded	1	0.0612	0.805
Temperature × Nutrient	2	2.522	0.0857
Temperature × Wounded	1	0.804	0.372
Nutrient × Wounded	2	0.346	0.708
Temperature × Nutrient × Wounded	2	0.0405	0.960

**Note:**

*P*-values defined as significant at a threshold of 0.05 are highlighted in bold.

### Response of microbial diversity to coral treatments

In addition to measuring changes in the responses of the host and algal symbionts, we also evaluated the coral-associated bacterial communities to fully assess the effects of these stressors on the holobiont. In summary, there were 428 unique bacterial OTUs across the entire rarefied dataset. On average, coral nubbins contained a low diversity of bacterial taxa, around 48.0 ± 4.0 unique OTUs. The most abundant family in the dataset was Endozoicomonadaceae with a mean relative abundance of 67.76% ± 3.51% and ranging from 2.90% to 99.44%. Only two samples under increased seawater temperature and ammonium enrichment, one intact and another scarred, contained less than 10% mean relative abundance of Endozoicomonadaceae. Although the Greengenes database identifies this family as Endozoicomonaceae, here the updated taxonomic assignment of Endozoicomonadaceae is used (Bartz et al., 2018; Neave et al., 2016). Other abundant families included Desulfovibrionaceae (5.51% ± 1.55% and ranging from 0.00% to 59.91%), Enterobacteriaceae (3.79% ± 1.39% and ranging from 0.00% to 76.73%), Rhodobacteraceae (5.88% ± 0.89% and ranging from 0.00% to 32.71%), and Moraxellaceae (2.83% ± 0.78% and ranging from 0.00% to 41.22%). On average, coral nubbins with ambient nutrients had a Chao1 index of 68.56 ± 6.06, although this did not differ with coral nubbins under ammonium or nitrate enrichment (*F* = 2.053, *P* = 0.14) which had indices of 47.37 ± 5.56 and 71.47 ± 12.51, respectively. Coral nubbins in ambient nutrient conditions also were not significantly different (*F* = 0.377, *P* = 0.69) via Simpson’s diversity (0.472 ± 0.069) when compared to ammonium (0.386 ± 0.055) or nitrate (0.446 ± 0.068).

**Figure 4:**
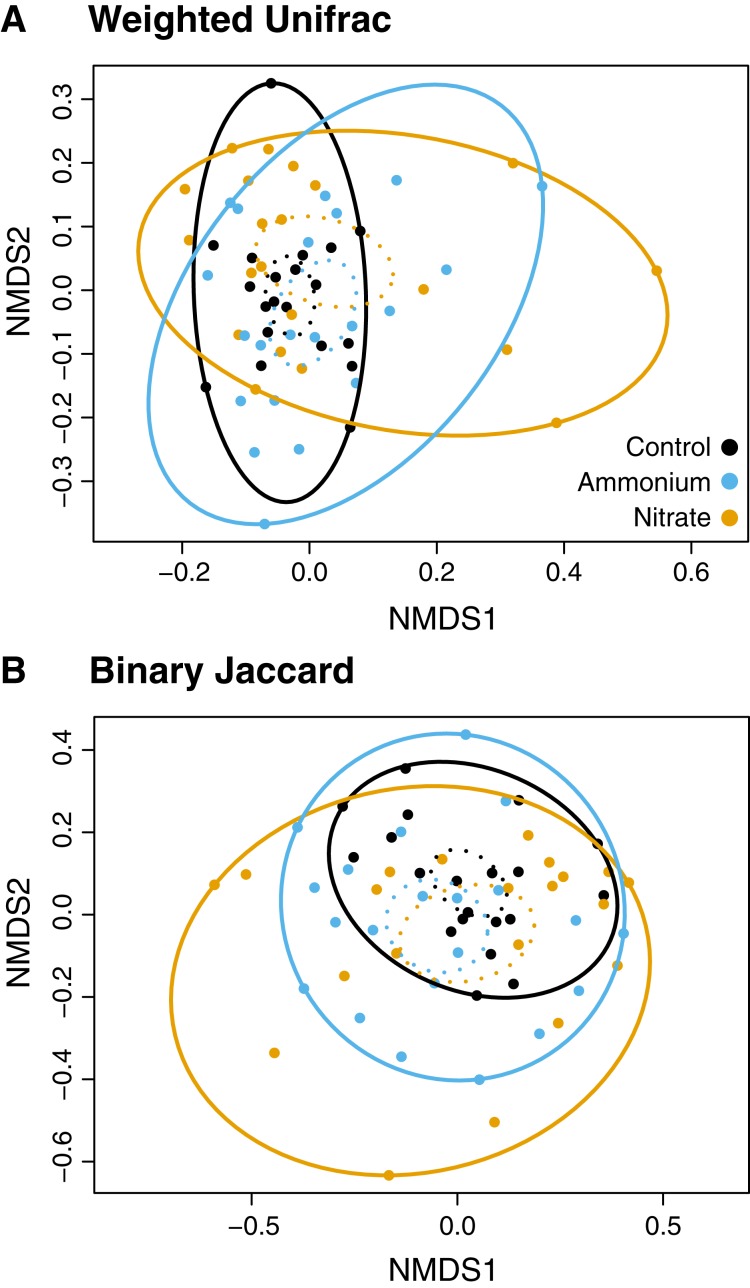
NMDS plots of the microbial community associated with nutrient treatment. Both dissimilarity measures, (A) Weighted Unifrac (*F* = 4.1, *P* < 0.05) and (B) Binary Jaccard (*F* = 4.2, *P* < 0.05), show increased community dispersion by nutrient treatment for the log-transformed OTU table (Table S7). Dashed ellipses designate standard errors of points with 95% confidence limit. Solid ellipses enclose all points within a group.

**Figure 5:**
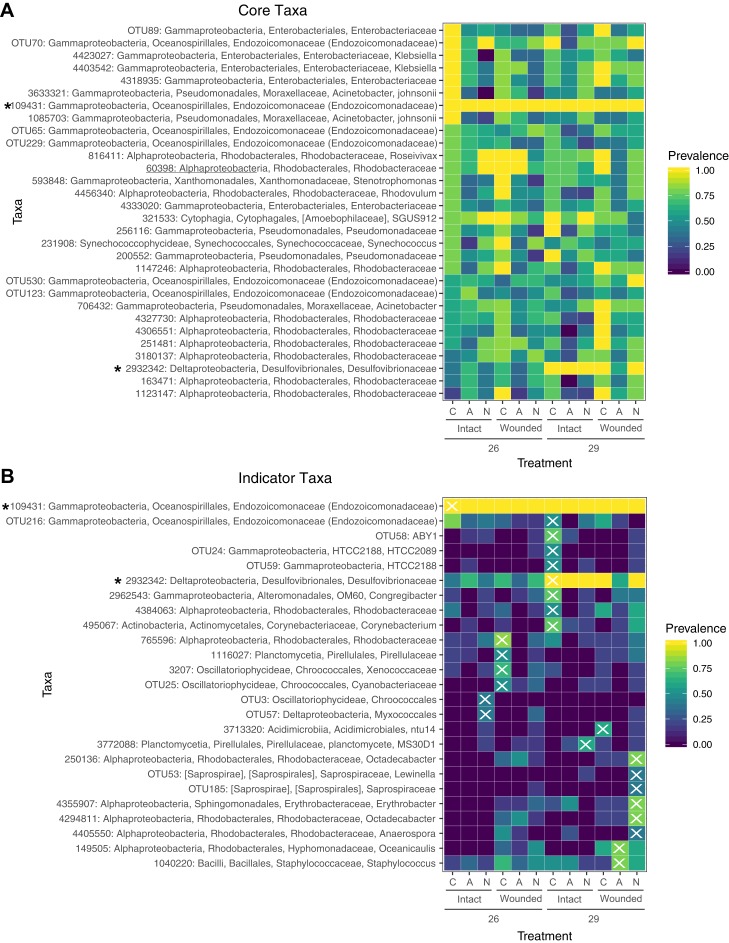
Prevalence of core and indicator microbial taxa by treatment. (A) The core microbiome consists of those OTUs that occur in at least 50% of the samples. (B) Indicator taxa were identified for treatment combinations with Indicator Species Analysis. OTUs were considered indicators for a treatment group if they had a significant indicator value and are designated with an X. Not all treatment combinations contain indicator taxa. Taxa marked with an asterisk are both core and indicators of a specific group. Prevalence is determined by the proportion of samples within a treatment group containing that OTU.

While there were no clear associations between alpha or beta diversity with Symbiodiniaceae densities, host growth rates, or host healing rates (Tables S5 and S6) there were differences in the microbial community structure across treatment regimes (Figs. 4 and 5). Differences in beta diversity between treatment groups were identified from the log-transformed community data. While PERMANOVA tests for distinct communities were significant for temperature, wounding, and nitrogen with various dissimilarity measures, all *R*^2^ values were less than 0.1 (Table S7). Therefore, these results were not considered representative of biologically distinct communities. No treatment interactions produced significantly distinct communities (Table S7). However, unlike community dissimilarity measures, there were significant differences between nitrogen treatment group dispersions for Binary Jaccard (PERMDISP, *F* = 4.210, *P* < 0.05) and Weighted Unifrac (PERMDISP, *F* = 4.140, *P* < 0.05) measures of community dissimilarity (Fig. 4; Table S8). Pairwise comparisons for associations showed that for the Binary Jaccard and Weighted Unifrac measures, corals under both nitrate and ammonium treatments were significantly more variable compared to coral microbiomes under ambient nutrients conditions (*P* < 0.05 and *P* < 0.05, respectively), but nitrate and ammonium were not significantly different from one another (*P* = 0.63 and *P* = 0.61, respectively). Group dispersions were also significantly different by temperature with the Binary Jaccard dissimilarity measures (PERMDISP, *F* = 6.730, *P* < 0.05, Fig. S2).

### Prevalence and associations of microbial taxa across treatments

A total of 30 OTUs were identified as comprising the core microbiome which were present in at least 50% of samples. Taxa in the core microbiome were evaluated for prevalence across samples in the different treatments (Fig. 5A). A single OTU (ID: 109431) of the family Endozoicomonadaceae was found in every sample (Fig. 5A). Eight OTUs of the families Moraxellaceae, Enterobacteriaceae, and Endozoicomonadaceae were found in every control sample. Nearly every coral sample at 29 °C contained an OTU in the family Desulfovibrionaceae (ID: 2932342), whereas this OTU was less prevalent in samples at 26 °C (Fig. 5A). OTUs from the family Rhodobacteraceae were prevalent in wounded coral samples at ambient nutrient levels, however, the specific Rhodobacteraceae species differed based on whether the sample had been exposed to seawater at 26 or 29 °C. OTUs in this family were also less prevalent in wounded coral samples exposed to nitrogen enrichment (Fig. 5A).

Using a rarefied OTU table, indicator species analysis identified associations between bacterial taxa and treatment combinations (Fig. 5B). A total of 25 OTUs were significant indicators with a *P* < 0.05, however, one of these OTUs had an indicator value < 0.5 (Table S9). The control group (ambient nutrients, intact, 26 °C) had a single indicator OTU of the family Endozoicomonadaceae. The indicator value of 0.344 for this OTU (Table S9) is likely due to the presence of this OTU in every sample (Figs. 5A and 5B). While this taxon dominates control corals with >95% relative abundance in all samples, it is not exclusive to the control group. Eight taxa were indicators for high temperature only, including an OTU of the family Desulfovibrionaceae which was also a member of the core microbiome (Fig. 5A). OTUs of the family Rhodobacteraceae were indicators for various treatment groups with wounding, high temperature, or with the combination of the two with nitrogen enrichment (Fig. 5B). The treatment groups of ammonium enrichment, ammonium or nitrate enrichment with wounding, and ammonium enrichment with high temperature did not have any significant indicator taxa (Fig. 5B).

## Discussion

Our study tested the hypothesis that different nitrogen (N) sources would have contrasting effects on *P. meandrina* growth, Symbiodiniaceae densities, wound healing, and the coral microbiome in response to seawater warming and simulated parrotfish corallivory. In contrast to our hypothesis, we observed that N source did not have divergent effects on the impacts of seawater warming and simulated corallivory on *P. meandrina* growth rates. However, N source did mediate the impacts of these stressors on Symbiodiniaceae densities and wound healing rates. In fact, intact and wounded corals were able to maintain growth rates under warmer temperatures and across nitrogen regimes. However, ammonium enrichment increased Symbiodiniaceae densities across temperature treatments. We also observed that warmer temperatures reduced tissue regeneration, but that ammonium enrichment counteracted this effect. At the microbial scale, community variability increased with nitrogen enrichment. We also observed distinct bacterial taxa that were indicators of corals under seawater warming, wounding, and the combination of these treatments with nitrogen enrichment.

### Potential trade-offs between growth and wound healing under seawater warming

Corals can experience a trade-off between metabolic processes (e.g., growth, gametogenesis) and tissue regeneration, which is often prioritized over coral growth (reviewed by Henry & Hart, 2005). Yet our study provides evidence that these tradeoffs are complex and depend on the abiotic conditions. The *P. meandrina* nubbins in our study maintained growth rates despite simulated corallivory under higher temperature and nitrogen enrichment. In contrast, Cameron & Edmunds (2014) found that simulated scraper corallivory decreased *P. meandrina* growth rates by ~42%. The discrepancies in results is perplexing given that our study had similar wound characteristics as those reported by Cameron & Edmunds (2014) and a similar experiment duration (21 days), but may be explained by differences in experimental approaches. Our experiment took place in mesocosms while Cameron & Edmunds (2014) conducted a field study in the back reef. Disparities in species responses can arise from different experimental approaches, and a response measured in the lab does not always translate to the field. However, the present study and others demonstrate that *P. meandrina* corals are able to maintain growth and calcification rates at ~29 °C (Medellin-Maldonado et al., 2016; Muehllehner & Edmunds, 2008). Moreover, Lenihan & Edmunds (2010) observed that injured *P. verrucosa* corals under seawater warming (~29 °C) outgrew intact conspecifics but had reduced tissue regeneration. These data are in agreement with our results showing that *P. meandrina* nubbins under seawater warming maintained growth (Fig. 1) but had lower wound healing rates (Fig. 2).

Wound healing for *P. meandrina* nubbins was ~66% lower at 29 °C than at 26 °C, suggesting that corals at warmer temperatures are less likely to recover from predation events. Seawater warming can reduce energy reserves in *Pocillopora* corals (Rodriguez-Troncoso, Carpizo-Ituarte & Cupul-Magana, 2010), which may explain the reduction in wound healing rates we observed. Pocilloporid corals may shift cellular resources to growth over tissue regeneration under warm water conditions. Evidence for this pattern has been observed in *Oculina patagonica* where growth is maintained with seawater warming while wound healing is suppressed (Serrano, Ribes & Coma, 2017). These patterns suggest that for *Pocillopora* spp. corals under warmer temperatures, a trade-off may exist between growth and tissue regeneration where growth is prioritized.

Nitrogen supply alleviated this trade-off by counteracting the effects of seawater warming on tissue regeneration rates. However, the effects of nitrogen likely depend on the concentration of nutrients. When considering ammonium enrichment alone, anthropogenic-driven concentrations (~20 µM) was shown to reduce coral wound healing rates (Koop et al., 2001). Yet we showed here that at naturally-occurring concentrations typical of fish excretion (~1–4 µM) (Holbrook et al., 2008; Meyer & Schultz, 1985; Shantz et al., 2015), ammonium supply can increase wound healing rates regardless of temperature (Fig. 2). This pattern indicates that *P. meandrina* may be more robust at recovering from predation events under warmer temperatures when nitrogen from fish excretion is readily available. Similarly, nitrate counteracted the effects of seawater warming on wound healing at 29 °C. In contrast, Renegar, Blackwelder & Moulding (2008) found that nitrate enrichment (~10 µM) reduced wound healing between ~10–60% depending on the coral species. The disparity in our findings can likely be explained by the lower nitrate concentrations used in our study (~4 µM), which are more environmentally relevant and thus less deleterious to corals. Altogether, our data suggest that *P. meandrina* corals prioritize growth over tissue regeneration under warm water conditions but that nitrogen supply can neutralize this effect.

### Seawater warming and nitrogen source mediate the effects on Symbiodiniaceae densities

A priori, we reasoned that Symbiodiniaceae densities would decrease under seawater warming and increase in response to nitrogen supply. However, under ambient nutrient conditions, seawater warming had little effect on Symbiodiniaceae densities. The lack of temperature effect is surprising given that Symbiodiniaceae densities tend to decline with seawater warming. For instance, Schmidt et al. (2016) found that seawater warming (~30 °C) reduces Symbiodiniaceae densities in *P. meandrina* corals. When seawater temperatures are approximately 30 °C in Moorea, Symbiodiniaceae densities for *P. meandrina* can also be reduced by upward of 35% (Putnam & Edmunds, 2011). Moreover, *Pocillopora* bleaching in Moorea has been observed for ~4.6 accumulated heat stress weeks (C°-weeks) when temperatures >29.0 °C (Pratchett et al., 2013). Such effects may not have been observed in our study due to its short duration (21 days) and that our warm water treatment did not exceed the 29.0 °C threshold required for *Pocillopora* bleaching in this region.

In regard to nitrogen supply, ammonium enrichment increased Symbiodiniaceae densities by ~30% compared to ambient conditions. Yet we did not observe significant changes in Symbiodiniaceae densities in response to nitrate. These results are in accordance with several studies showing that ammonium supply increases Symbiodiniaceae populations compared to corals without nitrogen enrichment, while nitrate enrichment tends to lower Symbiodiniaceae densities (Chase et al., 2018; Shantz & Burkepile, 2014). These trends may be due to the energetic costs of nitrate assimilation by Symbiodiniaceae for photosynthesis (Grover et al., 2003; Patterson et al., 2010).

We observed no effect of simulated corallivory wounds on Symbiodiniaceae densities compared to intact corals. This contradicts previous research showing that wounds decrease Symbiodiniaceae densities (Rotjan et al., 2006; Shirur, Jackson & Goulet, 2016). For example, Rotjan et al. (2006) found that parrotfish corallivory reduced Symbiodiniaceae densities of grazed *Orbicella* spp. in Belize. Simulated damage on gorgonians can also reduce Symbiodiniaceae densities in recovering tissues and tissues neighboring lesions (Shirur, Jackson & Goulet, 2016). The disparity in our observed results is likely attributable to the degree of damage. In our study, we simulated a single parrotfish scar on each coral nubbin while the coral colonies observed by Rotjan et al. (2006) typically had >30 parrotfish bites per colony. Thus, the degree of corallivory damage likely determines the impacts to Symbiodiniaceae populations.

### Varying N source increases microbial community variability and produces distinct indicator taxa

Although different nitrogen sources did not produce distinct microbial communities, microbial community dispersion increased significantly with the addition of nitrate or ammonium. Control corals under no stress had stable microbial communities with several core microbial members consistent in every sample (Fig. 5A). Similarly, corals under ambient nutrient regimes shared a degree of stability that was lost with the addition of ammonium or nitrate (Fig. 4). The addition of nitrogen increased sample to sample variability, suggesting a reduction in the host’s ability to regulate its microbial associates under stress (Zaneveld, McMinds & Vega Thurber, 2017). While our results suggest that microbiome composition changes under nitrogen enrichment stress, these changes are not deterministic and do not result in predictable stressed community states.

We also found that different stress regimes had indicator taxa that contribute to nitrogen cycling and may play an important role in regulating nitrogen availability in the host. In fact, nitrogen-fixation rates in the holobiont may moderate the hosts response to stress (Rädecker et al., 2015). For instance, the abundance of nitrogen fixing bacteria and total nitrogen fixation in the coral increases with higher temperatures (Cardini et al., 2016; Santos et al., 2014). It has been suggested that under increased nitrogen availability, nitrogen fixation rates would be reduced while nitrification and denitrification would increase to reduce internal nitrogen levels and maintain internal nitrogen limitation (Rädecker et al., 2015), which is necessary for a stable symbiosis with Symbiodiniaceae (Muscatine et al., 1989; Yellowlees, Rees & Leggat, 2008). However, one study found that both nitrogen-fixing and denitrifying bacteria in the coral *Acropora hemprichii* increased in response to increased nitrogen (Jessen et al., 2013). Likewise, bacteria of the order Chroococcales, notably *Cyanobacteria*, are known nitrogen-fixing taxa (Lesser, 2007; Wegley et al., 2007), and in the present study are indicators of wounded corals and corals exposed to excess nitrogen. Bacteria of the family Pirellulaceae are ammonium-oxidizers in sponges and may be conducting nitrification in corals (Gade et al., 2004; Kellogg, Ross & Brooke, 2016; Mohamed et al., 2010). Counterintuitively, taxa of this family are indicators of wounded corals and corals under high temperature and nitrate enrichment in the present study, rather than of ammonium enriched corals. While indicator species analysis of stress treatments identified several potential players in coral nitrogen metabolism, further functional studies are necessary to correlate community composition with changes in host nitrogen-cycling.

### Indicator taxa are characteristic of control and disturbed environmental regimes on reefs

Indicator taxa observed in this study support evidence of previous associations between bacterial taxa and holobiont stress (Maher et al., 2019; McDevitt-Irwin et al., 2017). Bacteria from the order Oceanspirillales are hypothesized to provide a beneficial function to the coral holobiont (Pantos et al., 2015), perhaps through their contribution to sulfur cycling (Raina et al., 2010). A single taxon from this order is an indicator of control corals and, although it is present in every sample, the relative abundance of this taxon decreases with stress (Maher et al., 2019). Another taxon from this order is also an indicator for high temperature along with a taxon from the order Actinomycetales (Fig. 5B), which is proposed to contain antibacterial properties (Mahmoud & Kalendar, 2016; Nithyanand, Manju & Pandian, 2011). These potentially beneficial taxa may moderate the host response to stress. Microbes associated with coral mucus have been hypothesized to produce antibiotic activities that select against potentially invasive microbes; however, antibiotic activity from these microbes is typically reduced during a period of high temperature (Ritchie, 2006). Therefore, any potential antibacterial properties of the coral tissue in the present study may be compromised in the high temperature treatment we applied here, although we can cannot confirm this hypothesis using these data alone.

Several indicator taxa also suggest increased opportunism in a community compromised with stress. A taxon from the family Desulfovibrionaceae is an indicator for high temperature and nearly all coral samples exposed to seawater at 29 °C contained this taxon (Fig. 5B). Desulfovibrionaceae is a sulfate-reducing bacterium (Bourne, Muirhead & Sato, 2011) and has been associated with increased seawater temperature and coral disease (Gajigan, Diaz & Conaco, 2017; Webster et al., 2011). Similarly, the family Saprospiraceae, which in the present study contains indicators of corals under nitrate enrichment, high temperature, and wounding, has been associated with corals exposed to fertilizer and municipal wastewater and other polluted environments (Jessen et al., 2013; Xia et al., 2008; Ziegler et al., 2016). Several taxa from the family Rhodobacteraceae were indicators of various treatments (Fig. 5B). These taxa are fast-growing and opportunistic (McDevitt-Irwin et al., 2017); however, the degree to which they proliferated with stress depends on the specific combination of stressors (Maher et al., 2019). While indicator species analysis can elucidate important patterns in bacterial associations, functional insights into consequences for the microbial community and host are limited. Additionally, further investigation is required to determine whether indicator taxa in a mesocosm experiment reflect indicator taxa on the reef. Of note, the identification of indicator or core microbiome members are limited here by the taxonomic resolution of the Greengenes database used here for taxonomic classification. For instance, the Greengenes database has not been updated since May 2013 while the SILVA v128 database was recently updated in 29/09/2016. Therefore, taxonomic classifications should utilize updated databases so as to avoid discarding sequences not annotated by an outdated database or with vague annotations suggesting contamination.

## Conclusions

As anthropogenic perturbations become more common on reefs, it is crucial to understand how these disturbances may change corals’ ability to cope with ongoing biotic processes. Corallivory is a common process on reefs that can exacerbate the response of corals to human impacts (Rice, Ezzat & Burkepile, 2019). The current study suggests that nitrogen source can alter the effects of concurrent seawater warming and corallivory on corals, while nitrogen enrichment can have distinct impacts on microbial community variability. Moreover, our results suggest that coral growth may be prioritized over tissue regeneration under warmer temperatures. However, how nitrogen availability and concentration may interact with corallivory and concurrent warming to drive changes to the coral microbial community warrants further research. Moreover, empirical studies are needed to understand how coral immune pathways involved in tissue regeneration respond to these anthropogenic stressors and across nutrient regimes. We observed increased microbiome variability with the addition of nitrogen and identified bacteria that are indicators of different stress regimes. Future research may investigate the functional capabilities of these indicator taxa, particularly in reference to nitrogen cycling, and how their function varies with anthropogenic forcing.

## Supplemental Information

10.7717/peerj.8056/supp-1 Supplemental Information 1 Schematic of experimental design. The experimental design crossing temperature (26 and 29 °C) and ammonium (NH_4_^+^) vs. nitrate (NO_3^−^_) nutrients with corallivory (closed circles: intact nubbins; open circles: wounded nubbins) (*n* = 2 mesocosms per treatment).

10.7717/peerj.8056/supp-2 Supplemental Information 2 NMDS plot of the microbial community associated with temperature treatment. The Binary Jaccard dissimilarity measure shows significantly different community dispersion by temperature for the log-transformed OTU table (*F* = 6.73, *P* < 0.05, Table S7). Dashed ellipses designate standard errors of points with 95% confidence limit. Solid ellipses enclose all points within a group.

10.7717/peerj.8056/supp-3 Supplemental Information 3 Microbial analysis mapping file after filtering. OTUs were filtered from the dataset if they (1) failed to align with PyNAST to the GreenGenes database, (2) were annotated as mitochondrial or chloroplast sequences, or (3) had less than 100 counts across the entire dataset. Next, samples with less than 1,000 reads were discarded (gray-colored sample rows).

10.7717/peerj.8056/supp-4 Supplemental Information 4 Linear mixed-effects model results for growth rate (mg cm^−2^ day^−1^) with Kenward-Roger approximation for degrees of freedom.

10.7717/peerj.8056/supp-5 Supplemental Information 5 Post hoc comparison results for the effects of temperature and nutrient on healing rate (mm^−2^ day^−1^).

10.7717/peerj.8056/supp-6 Supplemental Information 6 Post hoc comparison results for the effects of temperature and nutrients on Symbiodiniaceae densities (10^5^ cells cm^−2^).

10.7717/peerj.8056/supp-7 Supplemental Information 7 Effects of healing rate, Symbiodiniaceae density, and growth rate on microbial community alpha diversity metrics. Alpha diversity metrics (Chao1 index, Simpson’s index, Faith’s phylogenetic diversity) were regressed against host measurements using LMMs with host measurement (growth rate, healing rate, and Symbiodiniaceae density) as the fixed effect and tank and parent colony as random effects. Chao1 and Faith’s PD were log-transformed, while Simpson’s Index was arcsine-transformed to improve normality. *P*-values were approximated with the lmerTest package in R.

10.7717/peerj.8056/supp-8 Supplemental Information 8 Effects of healing rate, Symbiodiniaceae density, and growth rate on microbial community dissimilarity. PERMANOVA results for differences in community dissimilarity measured by four dissimilarity measures by host measurement.

10.7717/peerj.8056/supp-9 Supplemental Information 9 Effects of temperature, nutrients, wounding, and their interaction on microbial community dissimilarity. PERMANOVA results for differences between groups based on four dissimilarity measures.

10.7717/peerj.8056/supp-10 Supplemental Information 10 Effects of treatment on microbial community group dispersion. PERMDISP results for differences within treatments based on four dissimilarity measures.

10.7717/peerj.8056/supp-11 Supplemental Information 11 Indicator taxa by treatment combination. Indicator species analysis was conducted on the rarefied OTU table with a correction for unequal group sizes. Groups were defined as the treatment combination of temperature, nutrients, and wounding. Taxa with a significant indicator value are listed. Only treatment combinations with significant indicators are included.

## Additional Information and Declarations

### Field Study Permissions

The following information was supplied relating to field study approvals (i.e., approving body and any reference numbers): Research was completed under permits issued by the French Polynesian Government (Délégation à la Recherche) and the Haut-commissariat de la République en Polynésie Francaise (DTRT) (Protocole d’Accueil 2005–2018).

### Data Availability

The following information was supplied regarding data availability: Data is available at GitHub: https://github.com/mallorymrice/Poc_2016.git Microbial data is available at NCBI Sequence Read Archive under the BioProject ID PRJNA549489.

## References

[ref-1] Apprill A, McNally S, Parsons R, Weber L (2015). Minor revision to V4 region SSU rRNA 806R gene primer greatly increases detection of SAR11 bacterioplankton. Aquatic Microbial Ecology.

[ref-2] Bang C, Dagan T, Deines P, Dubilier N, Duschl WJ, Fraune S, Hentschel U, Hirt H, Hulter N, Lachnit T, Picazo D, Pita L, Pogoreutz C, Radecker N, Saad MM, Schmitz RA, Schulenburg H, Voolstra CR, Weiland-Brauer N, Ziegler M, Bosch TCG (2018). Metaorganisms in extreme environments: do microbes play a role in organismal adaptation?. Zoology.

[ref-3] Bartz J-O, Blom J, Busse H-J, Mvie JB, Hardt M, Schubert P, Wilke T, Goessmann A, Wilharm G, Bender J, Kämpfer P, Glaeser SP (2018). *Parendozoicomonas haliclonae* gen. nov. sp. nov. isolated from a marine sponge of the genus *Haliclona* and description of the family *Endozoicomonadaceae* fam. nov. comprising the genera *Endozoicomonas*, Parendozoicomonas, and *Kistimonas*. Systematic and Applied Microbiology.

[ref-4] Bates D, Machler M, Bolker BM, Walker SC (2015). Fitting linear mixed-effects models using lme4. Journal of Statistical Software.

[ref-5] Bourne DG, Morrow KM, Webster NS (2016). Insights into the coral microbiome: underpinning the health and resilience of reef ecosystems. Annual Review of Microbiology.

[ref-6] Bourne DG, Muirhead A, Sato Y (2011). Changes in sulfate-reducing bacterial populations during the onset of black band disease. ISME Journal.

[ref-7] Burkepile DE, Shantz AA, Adam TC, Munsterman KS, Speare KE, Ladd MC, Rice MM, Ezzat L, McIlroy S, Wong JC, Baker DM, Brooks AJ, Schmitt RJ, Holbrook SJ (2019). Nitrogen identity drives differential impacts of nutrients on coral bleaching and mortality. Ecosystems.

[ref-8] Cameron CM, Edmunds PJ (2014). Effects of simulated fish predation on small colonies of massive *Porites* spp. and *Pocillopora meandrina*. Marine Ecology Progress Series.

[ref-9] Caporaso JG, Bittinger K, Bushman FD, DeSantis TZ, Andersen GL, Knight R (2010a). PyNAST: a flexible tool for aligning sequences to a template alignment. Bioinformatics.

[ref-10] Caporaso JG, Kuczynski J, Stombaugh J, Bittinger K, Bushman FD, Costello EK, Fierer N, Pena AG, Goodrich JK, Gordon JI, Huttley GA, Kelley ST, Knights D, Koenig JE, Ley RE, Lozupone CA, McDonald D, Muegge BD, Pirrung M, Reeder J, Sevinsky JR, Tumbaugh PJ, Walters WA, Widmann J, Yatsunenko T, Zaneveld J, Knight R (2010b). QIIME allows analysis of high-throughput community sequencing data. Nature Methods.

[ref-11] Cardini U, Van Hoytema N, Bednarz VN, Rix L, Foster RA, Al-Rshaidat MMD, Wild C (2016). Microbial dinitrogen fixation in coral holobionts exposed to thermal stress and bleaching. Environmental Microbiology.

[ref-12] Chao A, Chiu C-H (2016). Species richness: estimation and comparison. Wiley StatsRef: Statistics Reference Online.

[ref-13] Chase TJ, Pratchett MS, Frank GE, Hoogenboom MO (2018). Coral-dwelling fish moderate bleaching susceptibility of coral hosts. PLOS ONE.

[ref-14] Cole AJ, Pratchett MS, Jones GP (2008). Diversity and functional importance of coral-feeding fishes on tropical coral reefs. Fish and Fisheries.

[ref-15] D’Angelo C, Wiedenmann J (2014). Impacts of nutrient enrichment on coral reefs: new perspectives and implications for coastal management and reef survival. Current Opinion in Environmental Sustainability.

[ref-16] Davies PS (1989). Short-term growth measurements of corals using an accurate buoyant weighing technique. Marine Biology.

[ref-17] De Caceres M, Legendre P (2009). Associations between species and groups of sites: indices and statistical inference. Ecology.

[ref-18] De Caceres M, Legendre P, Moretti M (2010). Improving indicator species analysis by combining groups of sites. Oikos.

[ref-19] Dufrêne M, Legendre P (1997). Species assemblages and indicator species: the need for a flexible asymmetrical approach. Ecological Monographs.

[ref-20] Edgar RC (2010). Search and clustering orders of magnitude faster than BLAST. Bioinformatics.

[ref-21] Edmunds PJ, LTER MCR (2018). MCR LTER: coral reef: long-term population and community dynamics: corals, ongoing since 2005.

[ref-22] Edmunds PJ, Leichter JJ, Johnston EC, Tong EJ, Toonen RJ (2016). Ecological and genetic variation in reef-building corals on four Society Islands. Limnology and Oceanography.

[ref-23] Ezzat L, Maguer JF, Grover R, Ferrier-Pages C (2015). New insights into carbon acquisition and exchanges within the coral–dinoflagellate symbiosis under NH4^+^ and NO3^−^ supply. Proceedings of the Royal Society B-Biological Sciences.

[ref-24] Fabricius KE, Dubinsky Z, Stambler N (2011). Factors determining the resilience of coral reefs to eutrophication: a review and conceptual model. Coral Reefs: An Ecosystem in Transition.

[ref-25] Fabricius KE, Cseke S, Humphrey C, De’ath G (2013). Does trophic status enhance or reduce the thermal tolerance of scleractinian corals? A review, experiment and conceptual framework. PLOS ONE.

[ref-26] Faith DP (1992). Conservation evaluation and phylogenetic diversity. Biological Conservation.

[ref-27] Ferrier-Pages C, Gattuso JP, Dallot S, Jaubert J (2000). Effect of nutrient enrichment on growth and photosynthesis of the zooxanthellate coral Stylophora pistillata. Coral Reefs.

[ref-28] Gade D, Schlesner H, Glockner FO, Amann R, Pfeiffer S, Thomm A (2004). Identification of planctomycetes with order-, genus-, and strain-specific 16S rRNA-targeted probes. Microbial Ecology.

[ref-29] Gajigan AP, Diaz LA, Conaco C (2017). Resilience of the prokaryotic microbial community of Acropora digitifera to elevated temperature. MicrobiologyOpen.

[ref-30] Glasl B, Herndl GJ, Frade PR (2016). The microbiome of coral surface mucus has a key role in mediating holobiont health and survival upon disturbance. ISME Journal.

[ref-31] Grover R, Maguer JF, Allemand D, Ferrier-Pages C (2003). Nitrate uptake in the scleractinian coral *Stylophora pistillata*. Limnology and Oceanography.

[ref-32] Heip CH, Herman PM, Soetaert K (1998). Indices of diversity and evenness. Oceanis.

[ref-33] Henry L-A, Hart M (2005). Regeneration from injury and resource allocation in sponges and corals - a review. International Review of Hydrobiology.

[ref-34] Holbrook SJ, Brooks AJ, Schmitt RJ, Stewart HL (2008). Effects of sheltering fish on growth of their host corals. Marine Biology.

[ref-35] Hughes TP, Anderson KD, Connolly SR, Heron SF, Kerry JT, Lough JM, Baird AH, Baum JK, Berumen ML, Bridge TC, Claar DC, Eakin CM, Gilmour JP, Graham NAJ, Harrison H, Hobbs J-PA, Hoey AS, Hoogenboom M, Lowe RJ, McCulloch MT, Pandolfi JM, Pratchett M, Schoepf V, Torda G, Wilson SK (2018). Spatial and temporal patterns of mass bleaching of corals in the Anthropocene. Science.

[ref-36] Jessen C, Lizcano JFV, Bayer T, Roder C, Aranda M, Wild C, Voolstra CR (2013). In-situ Effects of eutrophication and overfishing on physiology and bacterial diversity of the Red Sea Coral *Acropora hemprichii*. PLOS ONE.

[ref-37] Jokiel PL, Maragos JE, Frankzisket L, Stoddart DR, Johannes RE (1978). Coral growth: buoyant weight technique. Coral Reefs: Research Methods.

[ref-38] Kellogg CA, Ross SW, Brooke SD (2016). Bacterial community diversity of the deep-sea octocoral Paramuricea placomus. PeerJ.

[ref-39] Koop K, Booth D, Broadbent A, Brodie J, Bucher D, Capone D, Coll J, Dennison W, Erdmann M, Harrison P, Hoegh-Guldberg O, Hutchings P, Jones GB, Larkum AWD, O’Neil J, Steven A, Tentori E, Ward S, Williamson J, Yellowlees D (2001). ENCORE: The effect of nutrient enrichment on coral reefs. Synthesis of results and conclusions. Marine Pollution Bulletin.

[ref-40] Kozich JJ, Westcott SL, Baxter NT, Highlander SK, Schloss PD (2013). Development of a dual-index sequencing strategy and curation pipeline for analyzing amplicon sequence data on the MiSeq Illumina sequencing platform. Applied and Environmental Microbiology.

[ref-41] Lahti L, Sudarshan S, Tineka B, Jarkka S (2017). Tools for microbiome analysis in R. https://microbiome.github.io/tutorials/.

[ref-42] LaJeunesse TC, Parkinson JE, Gabrielson PW, Jeong HJ, Reimer JD, Voolstra CR, Santos SR (2018). Systematic Revision of symbiodiniaceae highlights the antiquity and diversity of coral endosymbionts. Current Biology.

[ref-43] Lenihan HS, Edmunds PJ (2010). Response of *Pocillopora verrucosa* to corallivory varies with environmental conditions. Marine Ecology Progress Series.

[ref-44] Lenth RV (2016). Least-squares means: the *R* package lsmeans. Journal of Statistical Software.

[ref-45] Lesser MP (2007). Coral reef bleaching and global climate change: can corals survive the next century?. Proceedings of the National Academy of Sciences of the United States of America.

[ref-46] Maher RL, Rice MM, McMinds R, Burkepile DE, Thurber RV (2019). Multiple stressors interact primarily through antagonism to drive changes in the coral microbiome. Scientific Reports.

[ref-47] Mahmoud HM, Kalendar AA (2016). Coral-associated actinobacteria: diversity, abundance, and biotechnological potentials. Frontiers in Microbiology.

[ref-48] Marubini F, Davies PS (1996). Nitrate increases zooxanthellae population density and reduces skeletogenesis in corals. Marine Biology.

[ref-49] Marubini F, Thake B (1999). Bicarbonate addition promotes coral growth. Limnology and Oceanography.

[ref-50] McDevitt-Irwin JM, Baum JK, Garren M, Vega Thurber RL (2017). Responses of coral-associated bacterial communities to local and global stressors. Frontiers in Marine Science.

[ref-51] McDonald D, Price MN, Goodrich J, Nawrocki EP, DeSantis TZ, Probst A, Andersen GL, Knight R, Hugenholtz P (2012). An improved Greengenes taxonomy with explicit ranks for ecological and evolutionary analyses of bacteria and archaea. ISME Journal.

[ref-52] McMurdie PJ, Holmes S (2013). phyloseq: an R package for reproducible interactive analysis and graphics of microbiome census data. PLOS ONE.

[ref-53] Medellin-Maldonado F, Cabral-Tena RA, Lopez-Perez A, Calderon-Aguilera LE, Norzagaray-Lopez CO, Chapa-Balcorta C, Zepeta-Vilchis RC (2016). Calcification of the main reef-building coral species on the Pacific coast of southern Mexico. Ciencias Marinas.

[ref-54] Meyer JL, Schultz ET (1985). Migrating haemulid fishes as a source of nutrients and organic matter on coral reefs. Limnology and Oceanography.

[ref-55] Meyer JL, Schultz ET, Helfman GS (1984). Fish schools: an asset to corals. Science.

[ref-56] Mohamed NM, Saito K, Tal Y, Hill RT (2010). Diversity of aerobic and anaerobic ammonia-oxidizing bacteria in marine sponges. ISME Journal.

[ref-57] Muehllehner N, Edmunds P (2008). Effects of ocean acidification and increased temperature on skeletal growth of two scleractinian corals, *Pocillopora meandrina* and Porites rus.

[ref-58] Muscatine L, Falkowski PG, Dubinsky Z, Cook PA, McCloskey LR (1989). The effect of external nutrient resources on the population-dynamics of zooxanthellae in a reef coral. Proceedings of the Royal Society B: Biological Sciences.

[ref-59] Muscatine L, Porter JW (1977). Reef corals: mutualistic symbioses adapted to nutrient-poor environments. BioScience.

[ref-60] Neave MJ, Apprill A, Ferrier-Pages C, Voolstra CR (2016). Diversity and function of prevalent symbiotic marine bacteria in the genus Endozoicomonas. Applied Microbiology and Biotechnology.

[ref-61] Nithyanand P, Manju S, Pandian SK (2011). Phylogenetic characterization of culturable actinomycetes associated with the mucus of the coral *Acropora digitifera* from Gulf of Mannar. FEMS Microbiology Letters.

[ref-62] Oksanen J, Kindt R, Legendre P, O’Hara B, Stevens MHH, Oksanen MJ, Suggests M (2007). The vegan package. Community Ecology Package.

[ref-63] Pantos O, Bongaerts P, Dennis PG, Tyson GW, Hoegh-Guldberg O (2015). Habitat-specific environmental conditions primarily control the microbiomes of the coral *Seriatopora hystrix*. ISME Journal.

[ref-64] Parada AE, Needham DM, Fuhrman JA (2016). Every base matters: assessing small subunit rRNA primers for marine microbiomes with mock communities, time series and global field samples. Environmental Microbiology.

[ref-65] Patterson K, Cakmak T, Cooper A, Lager I, Rasmusson AG, Escobar MA (2010). Distinct signalling pathways and transcriptome response signatures differentiate ammonium- and nitrate-supplied plants. Plant, Cell & Environment.

[ref-66] Pratchett MS, McCowan D, Maynard JA, Heron SF (2013). Changes in bleaching susceptibility among corals subject to ocean warming and recurrent bleaching in Moorea, French Polynesia. PLOS ONE.

[ref-67] Price MN, Dehal PS, Arkin AP (2010). FastTree 2 – approximately maximum-likelihood trees for large alignments. PLOS ONE.

[ref-68] Putnam HM, Edmunds PJ (2011). The physiological response of reef corals to diel fluctuations in seawater temperature. Journal of Experimental Marine Biology and Ecology.

[ref-69] R Development Core Team (2017). R: a language and environment for statistical computing.

[ref-70] Rädecker N, Pogoreutz C, Voolstra CR, Wiedenmann J, Wild C (2015). Nitrogen cycling in corals: the key to understanding holobiont functioning?. Trends in Microbiology.

[ref-71] Raina JB, Dinsdale EA, Willis BL, Bourne DG (2010). Do the organic sulfur compounds DMSP and DMS drive coral microbial associations?. Trends in Microbiology.

[ref-72] Renegar D-EA, Blackwelder P, Moulding AL (2008). Coral ultrastructural response to elevated pCO2 and nutrients during tissue repair and regeneration.

[ref-73] Rice MM, Ezzat L, Burkepile DE (2019). Corallivory in the Anthropocene: interactive effects of anthropogenic stressors and corallivory on coral reefs. Frontiers in Marine Science.

[ref-74] Rideout JR, He Y, Navas-Molina JA, Walters WA, Ursell LK, Gibbons SM, Chase J, McDonald D, Gonzalez A, Robbins-Pianka A, Clemente JC, Gilbert JA, Huse SM, Zhou HW, Knight R, Caporaso JG (2014). Subsampled open-reference clustering creates consistent, comprehensive OTU definitions and scales to billions of sequences. PeerJ.

[ref-75] Ritchie KB (2006). Regulation of microbial populations by coral surface mucus and mucus-associated bacteria. Marine Ecology Progress Series.

[ref-76] Rodriguez-Troncoso AP, Carpizo-Ituarte E, Cupul-Magana AL (2010). Differential response to cold and warm water conditions in *Pocillopora* colonies from the Central Mexican Pacific. Journal of Experimental Marine Biology and Ecology.

[ref-77] Rohwer F, Seguritan V, Azam F, Knowlton N (2002). Diversity and distribution of coral-associated bacteria. Marine Ecology Progress Series.

[ref-78] Rotjan RD, Dimond JL, Thornhill DJ, Leichter JJ, Helmuth B, Kemp DW, Lewis SM (2006). Chronic parrotfish grazing impedes coral recovery after bleaching. Coral Reefs.

[ref-79] Rotjan RD, Lewis SM (2008). Impact of coral predators on tropical reefs. Marine Ecology Progress Series.

[ref-80] Santos HF, Carmo FL, Duarte G, Dini-Andreote F, Castro CB, Rosado AS, Van Elsas JD, Peixoto RS (2014). Climate change affects key nitrogen-fixing bacterial populations on coral reefs. ISME Journal.

[ref-81] Schmidt GM, Wall M, Taylor M, Jantzen C, Richter C (2016). Large-amplitude internal waves sustain coral health during thermal stress. Coral Reefs.

[ref-82] Serrano E, Ribes M, Coma R (2017). Recurrent partial mortality events in winter shape the dynamics of the zooxanthellate coral *Oculina patagonica* at high latitude in the Mediterranean. Coral Reefs.

[ref-83] Shantz AA, Burkepile DE (2014). Context-dependent effects of nutrient loading on the coral–algal mutualism. Ecology.

[ref-84] Shantz AA, Ladd MC, Schrack E, Burkepile DE (2015). Fish-derived nutrient hotspots shape coral reef benthic communities. Ecological Applications.

[ref-85] Shirur KP, Jackson CR, Goulet TL (2016). Lesion recovery and the bacterial microbiome in two Caribbean gorgonian corals. Marine Biology.

[ref-86] Stimson J, Kinzie RA (1991). The temporal pattern and rate of release of zooxanthellae from the reef coral *Pocillopora damicornis* (Linnaeus) under nitrogen-enrichment and control conditions. Journal of Experimental Marine Biology and Ecology.

[ref-87] Vega Thurber RL, Burkepile DE, Fuchs C, Shantz AA, McMinds R, Zaneveld JR (2014). Chronic nutrient enrichment increases prevalence and severity of coral disease and bleaching. Global Change Biology.

[ref-88] Webster NS, Soo R, Cobb R, Negri AP (2011). Elevated seawater temperature causes a microbial shift on crustose coralline algae with implications for the recruitment of coral larvae. ISME Journal.

[ref-89] Wegley L, Edwards R, Rodriguez-Brito B, Liu H, Rohwer F (2007). Metagenomic analysis of the microbial community associated with the coral Porites astreoides. Environmental Microbiology.

[ref-90] Wickham H, Wickham H (2009). ggplot2 elegant graphics for data analysis introduction.

[ref-91] Xia Y, Kong YH, Thomsen TR, Nielsen PH (2008). Identification and ecophysiological characterization of epiphytic protein-hydrolyzing Saprospiraceae ("Candidatus epiflobacter" spp.) in activated sludge. Applied and Environmental Microbiology.

[ref-92] Yellowlees D, Rees TAV, Leggat W (2008). Metabolic interactions between algal symbionts and invertebrate hosts. Plant, Cell & Environment.

[ref-93] Zaneveld JR, Burkepile DE, Shantz AA, Pritchard CE, McMinds R, Payet JP, Welsh R, Correa AM, Lemoine NP, Rosales S, Fuchs C, Maynard JA, Vega Thurber R (2016). Overfishing and nutrient pollution interact with temperature to disrupt coral reefs down to microbial scales. Nature Communications.

[ref-94] Zaneveld JR, McMinds R, Vega Thurber R (2017). Stress and stability: applying the Anna Karenina principle to animal microbiomes. Nature Microbiology.

[ref-95] Zhou Z, Zhang GQ, Chen GM, Ni XZ, Guo LP, Yu XP, Xiao CL, Xu YL, Shi XW, Huang B (2017). Elevated ammonium reduces the negative effect of heat stress on the stony coral *Pocillopora damicornis*. Marine Pollution Bulletin.

[ref-96] Ziegler M, Roik A, Porter A, Zubier K, Mudarris MS, Ormond R, Voolstra CR (2016). Coral microbial community dynamics in response to anthropogenic impacts near a major city in the central Red Sea. Marine Pollution Bulletin.

[ref-97] Zuur A, Ieno E, Walker N, Saveliev A, Smith G, Gail M, Krickeberg K, Samet JM, Tsiatis A, Wong W (2009). Mixed effects models and extensions in ecology with R.

