# Supplementary figures and images for: Different nitrogen sources speed recovery from corallivory and uniquely alter the microbiome of a reef-building coral
Source: PeerJ. 2019 Nov 15;7:e8056. doi: 10.7717/peerj.8056 (PMC6859885; doi:10.7717/peerj.8056)

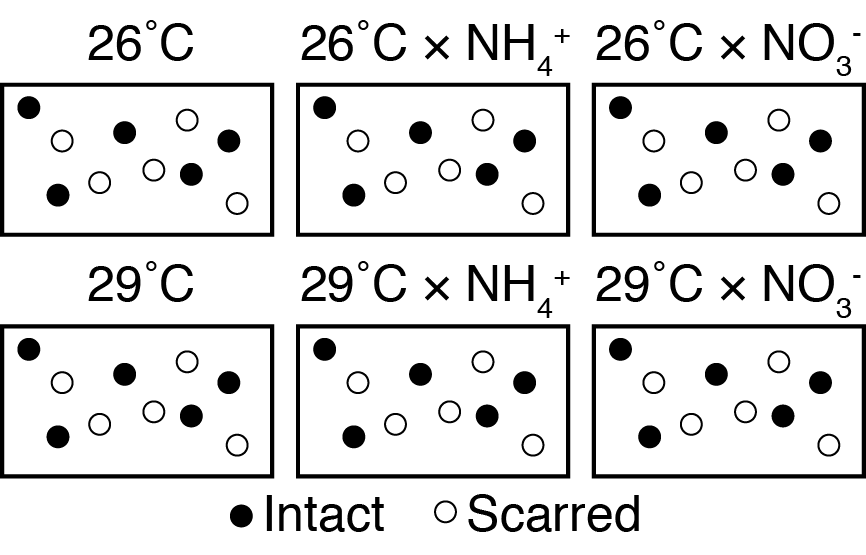

Supplement: Supplemental Information 1 — The experimental design crossing temperature (26 and 29 °C) and ammonium (NH4+) vs. nitrate (NO3−) nutrients with corallivory (closed circles: intact nubbins; open circles: wounded nubbins) (n = 2 mesocosms per treatment). [file peerj-07-8056-s001.png]

NMDS2

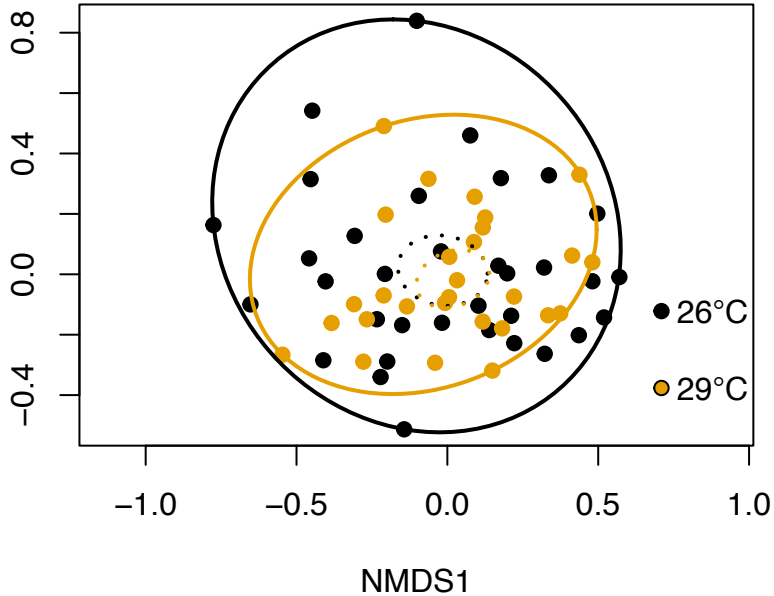

Supplement: Supplemental Information 2 — The Binary Jaccard dissimilarity measure shows significantly different community dispersion by temperature for the log-transformed OTU table (F = 6.73, P < 0.05, Table S7). Dashed ellipses designate standard errors of points with 95% confidence limit. Solid ellipses enclose all points within a group. [file peerj-07-8056-s002.pdf]
