# Supplementary material for: Different nitrogen sources speed recovery from corallivory and uniquely alter the microbiome of a reef-building coral
Source: PeerJ. 2019 Nov 15;7:e8056. doi: 10.7717/peerj.8056 (PMC6859885; doi:10.7717/peerj.8056)
Supplement: Supplemental Information 3 — OTUs were filtered from the dataset if they (1) failed to align with PyNAST to the GreenGenes database, (2) were annotated as mitochondrial or chloroplast sequences, or (3) had less than 100 counts across the entire dataset. Next, samples with less than 1,000 reads were discarded (gray-colored sample rows). [file peerj-07-8056-s003.docx]

**Table S1. Microbial analysis mapping file after filtering.** OTUs were filtered from the dataset if they 1) failed to align with PyNAST to the GreenGenes database, 2) were annotated as mitochondrial or chloroplast sequences, or 3) had less than 100 counts across the entire dataset. Next, samples with less than 1000 reads were discarded (grey-colored sample rows).

| **SampleID** | **Tank** | **Nutrient** | **Temperature** | **Scarring** | **Colony** | **Treatment** | **Stress** | **Seq Depth** |
| --- | --- | --- | --- | --- | --- | --- | --- | --- |
| m.ch.15 | D | Control | Control | Control | C5 | Control | none | 12930 |
| m.ch.14 | D | Control | Control | Control | C4 | Control | none | 40274 |
| m.ch.11 | D | Control | Control | Control | C1 | Control | none | 73948 |
| m.ch.19 | L | Control | Control | Control | C9 | Control | none | 4 |
| m.ch.18 | L | Control | Control | Control | C8 | Control | none | 11320 |
| m.ch.17 | L | Control | Control | Control | C7 | Control | none | 24526 |
| m.ch.35 | F | Control | High T | Control | C5 | High T | single | 250 |
| m.ch.34 | F | Control | High T | Control | C4 | High T | single | 22203 |
| m.ch.31 | F | Control | High T | Control | C1 | High T | single | 64340 |
| m.ch.39 | G | Control | High T | Control | C9 | High T | single | 585 |
| m.ch.38 | G | Control | High T | Control | C8 | High T | single | 4562 |
| m.ch.37 | G | Control | High T | Control | C7 | High T | single | 7570 |
| m.ch.21 | F | Control | High T | Scarred | C4 | High T, scarred | double | 5 |
| m.ch.24 | F | Control | High T | Scarred | C4 | High T, scarred | double | 8192 |
| m.ch.25 | F | Control | High T | Scarred | C5 | High T, scarred | double | 17118 |
| m.ch.27 | G | Control | High T | Scarred | C7 | High T, scarred | double | 1501 |
| m.ch.28 | G | Control | High T | Scarred | C8 | High T, scarred | double | 3083 |
| m.ch.29 | G | Control | High T | Scarred | C9 | High T, scarred | double | 26644 |
| m.ch.95 | E | NH4+ | Control | Control | C5 | NH4+ | single | 4602 |
| m.ch.94 | E | NH4+ | Control | Control | C4 | NH4+ | single | 24321 |
| m.ch.91 | E | NH4+ | Control | Control | C1 | NH4+ | single | 52777 |
| m.ch.98 | K | NH4+ | Control | Control | C8 | NH4+ | single | 8199 |
| m.ch.99 | K | NH4+ | Control | Control | C9 | NH4+ | single | 21860 |
| m.ch.97 | K | NH4+ | Control | Control | C7 | NH4+ | single | 24625 |
| m.ch.111 | C | NH4+ | High T | Control | C1 | NH4+, High T | double | 86 |
| m.ch.115 | C | NH4+ | High T | Control | C5 | NH4+, High T | double | 2859 |
| m.ch.114 | C | NH4+ | High T | Control | C4 | NH4+, High T | double | 7614 |
| m.ch.117 | J | NH4+ | High T | Control | C7 | NH4+, High T | double | 1 |
| m.ch.118 | J | NH4+ | High T | Control | C8 | NH4+, High T | double | 2151 |
| m.ch.119 | J | NH4+ | High T | Control | C9 | NH4+, High T | double | 5455 |
| m.ch.105 | C | NH4+ | High T | Scarred | C5 | NH4+, High T, scarred | triple | 1616 |
| m.ch.101 | C | NH4+ | High T | Scarred | C1 | NH4+, High T, scarred | triple | 17469 |
| m.ch.104 | C | NH4+ | High T | Scarred | C4 | NH4+, High T, scarred | triple | 20733 |
| m.ch.107 | J | NH4+ | High T | Scarred | C7 | NH4+, High T, scarred | triple | 4 |
| m.ch.108 | J | NH4+ | High T | Scarred | C8 | NH4+, High T, scarred | triple | 1962 |
| m.ch.109 | J | NH4+ | High T | Scarred | C9 | NH4+, High T, scarred | triple | 15391 |
| m.ch.84 | E | NH4+ | Control | Scarred | C4 | NH4+, scarred | double | 3058 |
| m.ch.85 | E | NH4+ | Control | Scarred | C5 | NH4+, scarred | double | 10134 |
| m.ch.81 | E | NH4+ | Control | Scarred | C1 | NH4+, scarred | double | 32454 |
| m.ch.89 | K | NH4+ | Control | Scarred | C9 | NH4+, scarred | double | 1691 |
| m.ch.88 | K | NH4+ | Control | Scarred | C8 | NH4+, scarred | double | 8458 |
| m.ch.87 | K | NH4+ | Control | Scarred | C7 | NH4+, scarred | double | 31459 |
| m.ch.54 | A | NO3- | Control | Control | C4 | NO3- | single | 8 |
| m.ch.55 | A | NO3- | Control | Control | C5 | NO3- | single | 4442 |
| m.ch.51 | A | NO3- | Control | Control | C1 | NO3- | single | 43207 |
| m.ch.57 | H | NO3- | Control | Control | C7 | NO3- | single | 13249 |
| m.ch.59 | H | NO3- | Control | Control | C9 | NO3- | single | 26058 |
| m.ch.58 | H | NO3- | Control | Control | C8 | NO3- | single | 27945 |
| m.ch.71 | B | NO3- | High T | Control | C1 | NO3-, High T | double | 0 |
| m.ch.74 | B | NO3- | High T | Control | C4 | NO3-, High T | double | 9623 |
| m.ch.75 | B | NO3- | High T | Control | C5 | NO3-, High T | double | 30273 |
| m.ch.79 | I | NO3- | High T | Control | C9 | NO3-, High T | double | 1070 |
| m.ch.77 | I | NO3- | High T | Control | C7 | NO3-, High T | double | 1139 |
| m.ch.78 | I | NO3- | High T | Control | C8 | NO3-, High T | double | 9360 |
| m.ch.61 | B | NO3- | High T | Scarred | C1 | NO3-, High T, scarred | triple | 5912 |
| m.ch.65 | B | NO3- | High T | Scarred | C5 | NO3-, High T, scarred | triple | 7723 |
| m.ch.64 | B | NO3- | High T | Scarred | C4 | NO3-, High T, scarred | triple | 10287 |
| m.ch.68 | I | NO3- | High T | Scarred | C8 | NO3-, High T, scarred | triple | 2 |
| m.ch.69 | I | NO3- | High T | Scarred | C9 | NO3-, High T, scarred | triple | 17725 |
| m.ch.67 | I | NO3- | High T | Scarred | C7 | NO3-, High T, scarred | triple | 23411 |
| m.ch.44 | A | NO3- | Control | Scarred | C4 | NO3-, scarred | double | 4904 |
| m.ch.45 | A | NO3- | Control | Scarred | C5 | NO3-, scarred | double | 21913 |
| m.ch.41 | A | NO3- | Control | Scarred | C1 | NO3-, scarred | double | 83601 |
| m.ch.47 | H | NO3- | Control | Scarred | C7 | NO3-, scarred | double | 9385 |
| m.ch.48 | H | NO3- | Control | Scarred | C8 | NO3-, scarred | double | 10564 |
| m.ch.49 | H | NO3- | Control | Scarred | C9 | NO3-, scarred | double | 15834 |
| m.ch.4 | D | Control | Control | Scarred | C4 | Scarred | single | 2334 |
| m.ch.1 | D | Control | Control | Scarred | C1 | Scarred | single | 5642 |
| m.ch.5 | D | Control | Control | Scarred | C5 | Scarred | single | 9368 |
| m.ch.9 | L | Control | Control | Scarred | C9 | Scarred | single | 3509 |
| m.ch.7 | L | Control | Control | Scarred | C7 | Scarred | single | 32292 |
| m.ch.8 | L | Control | Control | Scarred | C8 | Scarred | single | 59707 |
