# Supplementary material for: Different nitrogen sources speed recovery from corallivory and uniquely alter the microbiome of a reef-building coral
Source: PeerJ. 2019 Nov 15;7:e8056. doi: 10.7717/peerj.8056 (PMC6859885; doi:10.7717/peerj.8056)
Supplement: Supplemental Information 4 [file peerj-07-8056-s004.docx]

**Table S2. Linear mixed-effects model results for growth rate (mg cm^-2^ day^-1^) with Kenward-Roger approximation for degrees of freedom.**

| **Fixed Effects** | ***df*** | ***F*** | ***P*** |
| --- | --- | --- | --- |
| Temperature | 1 | 1.525 | 0.220 |
| Nutrient | 2 | 2.618 | 0.0782 |
| Wounded | 1 | 0.170 | 0.681 |
| Temperature × Nutrient | 2 | 1.228 | 0.298 |
| Temperature × Wounded | 1 | 2.219 | 0.140 |
| Nutrient × Wounded | 2 | 0.308 | 0.736 |
| Temperature × Nutrient × Wounded | 2 | 0.265 | 0.768 |
