# Supplementary material for: Different nitrogen sources speed recovery from corallivory and uniquely alter the microbiome of a reef-building coral
Source: PeerJ. 2019 Nov 15;7:e8056. doi: 10.7717/peerj.8056 (PMC6859885; doi:10.7717/peerj.8056)
Supplement: Supplemental Information 5 [file peerj-07-8056-s005.docx]

**Table S3. Post-hoc comparison results for the effects of temperature and nutrient on healing rate (mm^-2^ day^-1^).**

| **Comparison** | | ***β*** | | **SE** | | ***df*** | | ***t*** | ***P*** |
| --- | --- | --- | --- | --- | --- | --- | --- | --- | --- |
| 26ºC Control | 29ºC Control | | 1.107 | | 0.276 | | 46.0 | 4.017 | **0.0028** |
| 26ºC Control | 26ºC Ammonium | | 0.223 | | 0.276 | | 46.0 | 0.810 | 0.964 |
| 26ºC Control | 29ºC Ammonium | | 0.127 | | 0.276 | | 46.0 | 0.462 | 0.997 |
| 26ºC Control | 26ºC Nitrate | | 0.315 | | 0.276 | | 46.0 | 1.143 | 0.861 |
| 26ºC Control | 29ºC Nitrate | | 0.199 | | 0.270 | | 46.1 | 0.737 | 0.976 |
| 29ºC Control | 26ºC Ammonium | | -0.884 | | 0.276 | | 46.0 | -3.207 | **0.0277** |
| 29ºC Control | 29ºC Ammonium | | -0.980 | | 0.276 | | 46.0 | -3.554 | **0.0108** |
| 29ºC Control | 26ºC Nitrate | | -0.792 | | 0.276 | | 46.0 | -2.874 | 0.0635 |
| 29ºC Control | 29ºC Nitrate | | -0.908 | | 0.270 | | 46.1 | -3.364 | **0.0182** |
| 26ºC Ammonium | 29ºC Ammonium | | -0.0957 | | 0.276 | | 46.0 | -0.347 | 0.999 |
| 26ºC Ammonium | 26ºC Nitrate | | 0.0919 | | 0.276 | | 46.0 | 0.333 | 0.999 |
| 26ºC Ammonium | 29ºC Nitrate | | -0.0242 | | 0.270 | | 46.1 | -0.089 | 1 |
| 29ºC Ammonium | 26ºC Nitrate | | 0.188 | | 0.276 | | 46.0 | 0.681 | 0.983 |
| 29ºC Ammonium | 29ºC Nitrate | | 0.0716 | | 0.270 | | 46.1 | 0.265 | 0.999 |
| 26ºC Nitrate | 29ºC Nitrate | | -0.116 | | 0.270 | | 46.1 | -0.430 | 0.998 |

Notes: p-values defined as significant at a threshold of 0.05 are highlighted in bold.
