# Supplementary material for: Different nitrogen sources speed recovery from corallivory and uniquely alter the microbiome of a reef-building coral
Source: PeerJ. 2019 Nov 15;7:e8056. doi: 10.7717/peerj.8056 (PMC6859885; doi:10.7717/peerj.8056)
Supplement: Supplemental Information 6 [file peerj-07-8056-s006.docx]

**Table S4. Post-hoc comparison results for the effects of temperature and nutrients on Symbiodiniaceae densities (10^5^cells cm^-2^).**

| **Temperature Comparison** | | ***β*** | | **SE** | | ***df*** | | ***t*** | ***P*** |
| --- | --- | --- | --- | --- | --- | --- | --- | --- | --- |
| 26ºC | 29ºC | | -0.849 | | 0.302 | | 94.2 | -2.812 | **< 0.01** |
| **Nutrient Comparison** | | | ***β*** | | **SE** | | ***df*** | ***t*** | ***P*** |
| Control | Ammonium | | -1.085 | | 0.363 | | 94.1 | -2.993 | **< 0.01** |
| Control | Nitrate | | -0.415 | | 0.372 | | 94.4 | -1.115 | 0.508 |
| Ammonium | Nitrate | | 0.671 | | 0.375 | | 94.5 | 1.789 | 0.179 |

Notes: p-values defined as significant at a threshold of 0.05 are highlighted in bold.
