# Supplementary material for: Different nitrogen sources speed recovery from corallivory and uniquely alter the microbiome of a reef-building coral
Source: PeerJ. 2019 Nov 15;7:e8056. doi: 10.7717/peerj.8056 (PMC6859885; doi:10.7717/peerj.8056)
Supplement: Supplemental Information 7 — Alpha diversity metrics (Chao1 index, Simpson’s index, Faith’s phylogenetic diversity) were regressed against host measurements using LMMs with host measurement (growth rate, healing rate, and Symbiodiniaceae density) as the fixed effect and tank and parent colony as random effects. Chao1 and Faith’s PD were log-transformed, while Simpson’s Index was arcsine-transformed to improve normality. P-values were approximated with the lmerTest package in R. [file peerj-07-8056-s007.docx]

**Table S5. Effects of healing rate, Symbiodiniaceae density, and growth rate on microbial community alpha diversity metrics.** Alpha diversity metrics (Chao1 index, Simpson’s index, Faith’s phylogenetic diversity) were regressed against host measurements using LMMs with host measurement (growth rate, healing rate, and Symbiodiniaceae density) as the fixed effect and tank and parent colony as random effects. Chao1 and Faith's PD were log-transformed, while Simpson's Index was arcsine-transformed to improve normality. P-values were approximated with the lmerTest package in R.

| **Alpha diversity measure** | **Measurements** | ***β*** | **SE** | ***df*** | ***t*** | ***P*** |
| --- | --- | --- | --- | --- | --- | --- |
| **Chao1 index** | Healing rate | -0.152 | 0.203 | 23.794 | -0.748 | 0.462 |
|  | *Symbiodiniaceae* density | -0.721 | 0.862 | 55.783 | -0.836 | 0.407 |
|  | Growth rate | 0.008 | 0.004 | 53.525 | 1.991 | 0.051 |
| **Simpson’s diversity** | Healing rate | 0.016 | 0.088 | 24.901 | 0.179 | 0.859 |
|  | *Symbiodiniaceae* density | -0.375 | 0.413 | 58.000 | -0.907 | 0.368 |
|  | Growth rate | 0.0008 | 0.002 | 57.464 | 0.385 | 0.702 |
| **Faith’s phylogenetic diversity** | Healing rate | -0.235 | 0.158 | 25.000 | -1.484 | 0.150 |
|  | *Symbiodiniaceae* density | -0.828 | 0.693 | 49.518 | -1.194 | 0.238 |
|  | Growth rate | 0.005 | 0.003 | 53.674 | 1.576 | 0.121 |
