# Supplementary material for: Different nitrogen sources speed recovery from corallivory and uniquely alter the microbiome of a reef-building coral
Source: PeerJ. 2019 Nov 15;7:e8056. doi: 10.7717/peerj.8056 (PMC6859885; doi:10.7717/peerj.8056)
Supplement: Supplemental Information 8 — PERMANOVA results for differences in community dissimilarity measured by four dissimilarity measures by host measurement. [file peerj-07-8056-s008.docx]

**Table S6. Effects of healing rate, Symbiodiniaceae density, and growth rate on microbial community dissimilarity.** PERMANOVA results for differences in community dissimilarity measured by four dissimilarity measures by host measurement.

| **Dissimilarity Measure** | **Measurements** | ***df*** | **SS** | ***F*** | **R²** | ***P*** |
| --- | --- | --- | --- | --- | --- | --- |
| **Bray Curtis** | Healing rate | 1 | 0.230 | 0.776 | 0.029 | 0.873 |
|  | *Symbiodinium* density | 1 | 0.406 | 1.369 | 0.023 | 0.092 |
|  | Growth rate | 1 | 0.030 | 0.979 | 0.017 | 0.489 |
| **Binary Jaccard** | Healing rate | 1 | 0.319 | 0.844 | 0.032 | 0.883 |
|  | *Symbiodinium* density | 1 | 0.517 | 1.342 | 0.023 | **<0.05** |
|  | Growth rate | 1 | 0.377 | 0.971 | 0.016 | 0.552 |
| **Weighted Unifrac** | Healing rate | 1 | 0.089 | 0.988 | 0.037 | 0.456 |
|  | *Symbiodinium* density | 1 | 0.104 | 1.115 | 0.018 | 0.281 |
|  | Growth rate | 1 | 0.085 | 0.905 | 0.015 | 0.500 |
| **Unweighted Unifrac** | Healing rate | 1 | 0.286 | 1.198 | 0.044 | 0.197 |
|  | *Symbiodinium* density | 1 | 0.307 | 1.225 | 0.021 | 0.174 |
|  | Growth rate | 1 | 0.274 | 1.093 | 0.019 | 0.302 |

Notes: p-values defined as significant at a threshold of 0.05 are highlighted in bold.
