# Supplementary material for: Different nitrogen sources speed recovery from corallivory and uniquely alter the microbiome of a reef-building coral
Source: PeerJ. 2019 Nov 15;7:e8056. doi: 10.7717/peerj.8056 (PMC6859885; doi:10.7717/peerj.8056)
Supplement: Supplemental Information 9 — PERMANOVA results for differences between groups based on four dissimilarity measures. [file peerj-07-8056-s009.docx]

**Table S7. Effects of temperature, nutrients, wounding, and their interaction on microbial community dissimilarity.** PERMANOVA results for differences between groups based on four dissimilarity measures.

| **Dissimilarity Measure** | **Factor** | ***df*** | **SS** | ***F*** | **R²** | ***P*** |
| --- | --- | --- | --- | --- | --- | --- |
| **Bray Curtis** | Temperature | 1 | 0.492 | 1.723 | 0.0271 | **<0.05** |
|  | Wounding | 1 | 0.537 | 1.880 | 0.030 | **<0.05** |
|  | Nutrient | 2 | 0.695 | 1.218 | 0.038 | 0.108 |
|  | Temp × Wounded | 1 | 0.277 | 0.971 | 0.015 | 0.486 |
|  | Temp × Nutrient | 2 | 0.668 | 1.170 | 0.037 | 0.150 |
|  | Wounded × Nutrient | 2 | 0.599 | 1.04 | 0.033 | 0.324 |
|  | Temperature × Wounded × Nutrient | 2 | 0.642 | 1.12 | 0.035 | 0.192 |
| **Binary Jaccard** | Temperature | 1 | 0.462 | 1.217 | 0.019 | 0.064 |
|  | Wounded | 1 | 0.592 | 1.561 | 0.025 | **<0.01** |
|  | Nutrient | 2 | 0.956 | 1.261 | 0.040 | **<0.05** |
|  | Temp × Wounded | 1 | 0.365 | 0.964 | 0.015 | 0.589 |
|  | Temp × Nutrient | 2 | 0.869 | 1.147 | 0.037 | 0.087 |
|  | Wounded × Nutrient | 2 | 0.775 | 1.022 | 0.033 | 0.358 |
|  | Temperature × Wounded × Nutrient | 2 | 0.770 | 1.016 | 0.032 | 0.393 |
| **Weighted Unifrac** | Temperature | 1 | 0.146 | 1.563 | 0.025 | 0.098 |
|  | Wounded | 1 | 0.177 | 1.893 | 0.030 | **<0.05** |
|  | Nutrient | 2 | 0.252 | 1.345 | 0.043 | 0.144 |
|  | Temp × Wounded | 1 | 0.042 | 0.451 | 0.007 | 0.974 |
|  | Temp × Nutrient | 2 | 0.237 | 1.262 | 0.040 | 0.156 |
|  | Wounded × Nutrient | 2 | 0.152 | 0.813 | 0.025 | 0.712 |
|  | Temperature × Wounded × Nutrient | 2 | 0.206 | 1.099 | 0.035 | 0.317 |
| **Unweighted Unifrac** | Temperature | 1 | 0.401 | 1.640 | 0.026 | **<0.05** |
|  | Wounded | 1 | 0.400 | 1.637 | 0.026 | **<0.05** |
|  | Nutrient | 2 | 0.669 | 1.369 | 0.043 | **<0.05** |
|  | Temp × Wounded | 1 | 0.186 | 0.763 | 0.012 | 0.855 |
|  | Temp × Nutrient | 2 | 0.623 | 1.276 | 0.040 | 0.069 |
|  | Wounded × Nutrient | 2 | 0.460 | 0.942 | 0.029 | 0.602 |
|  | Temperature × Wounded × Nutrient | 2 | 0.566 | 1.158 | 0.037 | 0.185 |

Notes: p-values defined as significant at a threshold of 0.05 are highlighted in bold.
