# Supplementary material for: Different nitrogen sources speed recovery from corallivory and uniquely alter the microbiome of a reef-building coral
Source: PeerJ. 2019 Nov 15;7:e8056. doi: 10.7717/peerj.8056 (PMC6859885; doi:10.7717/peerj.8056)
Supplement: Supplemental Information 10 — PERMDISP results for differences within treatments based on four dissimilarity measures. [file peerj-07-8056-s010.docx]

**Table S8. Effects of treatment on microbial community group dispersion.** PERMDISP results for differences within treatments based on four dissimilarity measures.

| **Dissimilarity Measure** | **Factor** | ***df*** | **SS** | ***F*** | ***P*** |
| --- | --- | --- | --- | --- | --- |
| **Bray Curtis** | Temperature | 1 | 0.002 | 0.733 | 0.396 |
|  | Nutrient | 2 | 0.013 | 2.834 | 0.067 |
|  | Wounding | 1 | 0.0001 | 0.027 | 0.871 |
|  | Colony | 5 | 0.022 | 1.512 | 0.201 |
|  | Tank | 11 | 0.038 | 0.721 | 0.713 |
| **Binary Jaccard** | Temperature | 1 | 0.010 | 6.730 | **<0.05** |
|  | Nutrient | 2 | 0.014 | 4.209 | **<0.05** |
|  | Wounding | 1 | 0.001 | 0.899 | 0.347 |
|  | Colony | 5 | 0.006 | 0.622 | 0.683 |
|  | Tank | 11 | 0.023 | 0.711 | 0.722 |
| **Weighted Unifrac** | Temperature | 1 | 0.018 | 2.970 | 0.090 |
|  | Nutrient | 2 | 0.047 | 4.136 | **<0.05** |
|  | Wounding | 1 | 0.002 | 0.307 | 0.582 |
|  | Colony | 5 | 0.002 | 0.329 | 0.893 |
|  | Tank | 11 | 0.086 | 1.103 | 0.379 |
| **Unweighted Unifrac** | Temperature | 1 | 0.013 | 3.21 | 0.078 |
|  | Nutrient | 2 | 0.026 | 3.080 | 0.053 |
|  | Wounding | 1 | 0.005 | 1.004 | 0.320 |
|  | Colony | 5 | 0.013 | 0.570 | 0.722 |
|  | Tank | 11 | 0.039 | 0.498 | 0.895 |

Notes: p-values defined as significant at a threshold of 0.05 are highlighted in bold.
