# Supplementary material for: Different nitrogen sources speed recovery from corallivory and uniquely alter the microbiome of a reef-building coral
Source: PeerJ. 2019 Nov 15;7:e8056. doi: 10.7717/peerj.8056 (PMC6859885; doi:10.7717/peerj.8056)
Supplement: Supplemental Information 11 — Indicator species analysis was conducted on the rarefied OTU table with a correction for unequal group sizes. Groups were defined as the treatment combination of temperature, nutrients, and wounding. Taxa with a significant indicator value are listed. Only treatment combinations with significant indicators are included. [file peerj-07-8056-s011.docx]

**Table S9. Indicator taxa by treatment combination.** Indicator species analysis was conducted on the rarefied OTU table with a correction for unequal group sizes. Groups were defined as the treatment combination of temperature, nutrients, and wounding. Taxa with a significant indicator value are listed. Only treatment combinations with significant indicators are included.

| **Indicator taxa** | **Class** | **Order** | **Family** | **Indicator value** | ***P*** |
| --- | --- | --- | --- | --- | --- |
| **Control** |  |  |  |  |  |
| 109431 | Gammaproteobacteria | Oceanospirillales | Endozoicimonaceae | 0.344 | **<0.01** |
| **High Temperature – 29ºC** | |  |  |  |  |
| OTU216 | Gammaproteobacteria | Oceanospirillales | Endozoicimonaceae | 0.707 | **<0.05** |
| OTU58 | ABY1 |  |  | 0.685 | **<0.01** |
| OTU24 | Gammaproteobacteria | HTCC2188 | HTCC2089 | 0.637 | **<0.05** |
| OTU59 | Gammaproteobacteria | HTCC2188 | NA | 0.632 | **<0.05** |
| 2932342 | Deltaproteobacteria | Desulfovibrionales | Desulfovibrionaceae | 0.63 | **<0.05** |
| 2962543 | Gammaproteobacteria | Alteromonadales | OM60 | 0.619 | **<0.05** |
| 4384063 | Alphaproteobacteria | Rhodobacterales | Rhodobacteraceae | 0.559 | **<0.05** |
| 495067 | Actinobacteria | Actinomycetales | Corynebacteriaceae | 0.525 | **<0.05** |
| **Wounded** |  |  |  |  |  |
| 765596 | Alphaproteobacteria | Rhodobacterales | Rhodobacteraceae | 0.654 | **<0.05** |
| 1116027 | Planctomycetia | Pirellulales | Pirellulaceae | 0.62 | **<0.05** |
| 3207 | Oscillatoriophycideae | Chroococcales | Xenococcaceae | 0.598 | **<0.05** |
| OTU25 | Oscillatoriophycideae | Chroococcales | Cyanobacteriaceae | 0.571 | **<0.05** |
| **Nitrate** |  |  |  |  |  |
| OTU3 | Oscillatoriophycideae | Chroococcales | NA | 0.632 | **<0.05** |
| OTU57 | Deltaproteobacteria | Myxococcales | NA | 0.602 | **<0.05** |
| **High Temperature – 29ºC, Wounded** | | |  |  |  |
| 3713320 | Acidimicrobiia | Acidimicrobiales | ntu14 | 0.725 | **<0.01** |
| **High Temperature – 29ºC, Nitrate** | | |  |  |  |
| 3772088 | Planctomycetia | Pirellulales | Pirellulaceae | 0.679 | **<0.01** |
| **High Temperature – 29ºC, Nitrate, Wounded** | | |  |  |  |
| 250136 | Alphaproteobacteria | Rhodobacterales | Rhodobacteraceae | 0.701 | **<0.01** |
| OTU53 | [Saprospirae] | [Saprospirales] | Saprospiraceae | 0.632 | **<0.05** |
| OTU185 | [Saprospirae] | [Saprospirales] | Saprospiraceae | 0.575 | **<0.05** |
| 4355907 | Alphaproteobacteria | Sphingomonadales | Erythrobacteraceae | 0.575 | **<0.05** |
| 4294811 | Alphaproteobacteria | Rhodobacterales | Rhodobacteraceae | 0.543 | **<0.05** |
| 4405550 | Alphaproteobacteria | Rhodobacterales | Rhodobacteraceae | 0.527 | **<0.05** |
| **High Temperature – 29ºC, Ammonium, Wounded** | | |  |  |  |
| 149505 | Alphaproteobacteria | Rhodobacterales | Hyphomonadaceae | 0.618 | **<0.05** |
| 1040220 | Bacilli | Bacillales | Staphylococcaceae | 0.576 | **<0.05** |
